# Predicting food prices in Kenya using machine learning: a hybrid model approach with XGBoost and gradient boosting

**DOI:** 10.3389/frai.2025.1661989

**Published:** 2025-10-24

**Authors:** Benard O. Ogol, Evans Omondi, John Olukuru, Betsy Muriithi, Kennedy Senagi

**Affiliations:** ^1^@ilabAfrica, Strathmore University, Nairobi, Kenya; ^2^Institute of Mathematical Sciences, Strathmore University, Nairobi, Kenya; ^3^African Population and Health Research Center, Nairobi, Kenya; ^4^International Centre of Insect Physiology and Ecology, Nairobi, Kenya

**Keywords:** agricultural stakeholders, food insecurity, machine learning, malnutrition, policymakers, volatility

## Abstract

**Introduction:**

Food price volatility continues to be a significant concern in Kenya's economic development, posing challenges to the country's economic stability.

**Methodology:**

This study examines the application of machine learning methods, employing a hybrid approach that combines XGBoost and gradient boosting, to predict food prices in Kenya. The food prices data from the World Food Programme, covering the period from January 2006 to September 2024, as well as currency exchange rates data from the Central Bank of Kenya in US dollars (USD) and inflation rates data, were collated and preprocessed to be ready for analytics and machine learning. The augmented data were preprocessed and transformed, then used to train XGBoost, gradient boosting, LightGBM, decision tree, random forest, and linear regression. A hybrid model was then developed by stacking XGBoost and gradient boosting as the base models, with linear regression serving as the meta-model used to combine their predictions.

**Results:**

This model was then tuned using the hyperparameter random search method, achieving a mean absolute error of 0.1050, a mean squared error of 0.0261, a root mean square error of 0.1615, and an R-squared value of 0.9940, thereby surpassing the performance of all standalone models. We then applied cross-validation using 5-fold cross-validation and Diebold-Mariano tests to check for model overfitting and to perform model superiority analysis. Feature importance analysis using SHapley Additive exPlanations (SHAP) revealed that intuitive features influencing food prices are unit quantity, price type, commodity, and currency, while geographical factors such as county have a lesser impact. Finally, the model and its important features were saved as pickle files to facilitate the deployment of the model on a web application for food price predictions.

**Discussion:**

This data-driven decision support system can help policymakers and agricultural stakeholders (such as the Kenyan government) plan for future trends in food prices, potentially helping to prevent food insecurity in Kenya.

## 1 Introduction

Food is a fundamental necessity for human survival, significantly impacting the health, productivity, and overall well-being of humans. As part of achieving socioeconomic stability, the provision of abundant, affordable, and nutritious food remains a pressing issue. The volatility of food prices is now a major issue worldwide, as it impacts various areas, including financial stability, causing instability in macroeconomic conditions and affecting income, making it difficult to purchase food products (Gizaw and Myrland, 2025). The fluctuation of food prices has led to increased malnutrition among many individuals. A large number of these people come from countries affected by conflicts, causing relocation and destruction of property (Vos et al., 2020). The Food and Agriculture Organization projected that the food price crisis of 2007–2008 would lead to an increase in the number of malnourished people, which, according to the report, rose to 240 million people in Africa alone by 2008 (Abdallah et al., 2021). Similarly, Kalkuhl et al. (2016) noted that fluctuating food prices significantly impact the stability aspect of food and nutrition security, emphasizing that a price shock increase could result in inadequate nutrient intake, thereby affecting health and economic development. As stated by Alam et al. (2014) and Kalkuhl et al. (2015), fluctuations in food prices affect households' ability to purchase basic necessities, and this impact is equally distributed among low-income households and the rich. Consequently, a large portion of the disposable income of low-income households is used to meet their food needs due to the rise in food prices, which is a significant burden for them (Were et al., 2023). Attílio (2024) argued that volatility drivers, such as differences in international food prices and geopolitical tensions, continue to create uncertainty in food prices, even in the face of various interventions aimed at reducing price variation.

The situation is more severe in developing countries that rely heavily on food imports; therefore, market disturbances cause a rise in the prices of staple foods such as wheat, maize, rice, and vegetable oils (Korir et al., 2020). As stated by Vijayan et al. (2025), food availability alone does not ensure accessibility and reliability, as high prices, inefficient supply chains, and unaffordability persist. The price increase discourages investment in agriculture, thus limiting the increase in food production and disrupting the entire food supply chain (Headey and Fan, 2010). In addition, economic disturbances such as fluctuations in oil prices, disturbances in world markets, and changes in demand lead to further instability of food prices (Tadesse et al., 2014). In East Africa, volatility in staple food prices often undermines food security. For example, Tanzanian maize prices exhibit high seasonality, with annual fluctuations averaging 26.6%, which is significantly higher than the global average (Minot, 2014). In Uganda, spatial pricing has been attributed to market inefficiencies and infrastructure constraints (Shinyekwa and Ijjo, 2016). Similarly, however, econometric evidence from Rwanda has shown that regional markets for major staples, including bananas and potatoes, are co-integrated. Therefore, local shocks through the impacts of the trade network could quickly propagate from another region (Tesfaye and Gebremariam, 2020). Prices have remained highly volatile, even beyond that level, especially in the Sub-Saharan region.

Growing empirical evidence has strengthened the ability to model and predict food prices, particularly within low- and middle-income countries such as Kenya. Mutwiri (2019) applied the Seasonal Autoregressive Integrated Moving Average (SARIMA) model to forecast wholesale prices of tomatoes in Nairobi. The model exhibited a root mean square error (RMSE) of 32.063, a mean absolute percentage error (MAPE) of 125.251, and a mean absolute error (MAE) of 22.3, making it suitable for forecasting prices in this case. Similarly, econometric models such as SARIMA and ARIMA have also been used for food price forecasting in sub-Saharan countries, particularly in Kenya. Wanjuki et al. (2021) applied SARIMA models to Kenya's food and beverage CPI, achieving a mean absolute error (MAE) of 2.00%, a mean absolute percentage error (MAPE) of 1.62%, and a mean absolute scaled error (MASE) of 0.87%, capturing the seasonal variation effectively. Jayne et al. (2006) used VAR models on Kenyan maize markets, showing improved measures of adequacy (R^2^ > 0.70) when incorporating policy shocks.

These methods give good starting points but cannot effectively model nonlinearities and complex interactions between features. Sapakova et al. (2023) used decision trees, random forests, and gradient boosting in a study in Kazakhstan to predict food prices, where the random forest model emerged as the best, with the highest R^2^ score of 0.99, outperforming the other models. In a recent study, Nasir et al. (2025) introduced a hybrid forecasting framework that combines local mean decomposition, a progressive integrated moving average, and machine learning methods such as XGBoost, random forest, artificial neural networks (ANN), and support vector machines (SVMs); the framework was tested in relation to long-horizon financial time series. When applied to the National Association of Securities Dealers Automated Quotations (NASDAQ) Composite Index, however, the hybrid model proved to be the best among methods in terms of accuracy measures such as RMSE, MAE, MAPE, and the Diebold-Mariano-Statistic, which indicates the importance of decompression-based filtering coupled with ensemble learning when working toward a strong financial forecast. In addition, Zhang et al. (2025) used light gradient boosting models as the base model to forecast the prices of fresh farm produce such as bananas. The performance of the model was then compared with numerous other machine learning models, time series models, and artificial neural networks, from which LightGBM emerged to be the best in terms of accuracy and prediction with a mean square error of 0.0924, a mean absolute percentage error of 1.5234, and a mean square error of 0.0087 to predict prices of bananas, beef, and crucian carp.

Machine learning algorithms have been applied in various domains to support decision-making (Senagi et al., 2017; Muinde et al., 2023; Katchali et al., 2024; Kyalo et al., 2024, 2025). In this context, Mamoudan et al. (2022) presented an optimized model that integrates a convolutional neural network (CNN), long short-term memory (LSTM), and a genetic algorithm (GA) to forecast prices for time-sensitive goods. This model initially determines the prices of rival goods using a game theory model. The suggested model was evaluated, achieving a mean squared error of 0.0023, a mean absolute error of 0.0396, and an RMSE of 0.05. The R^2^ value of the suggested algorithm is 0.9378. The accuracy of food price prediction models depends on the careful selection of input variables that reflect both supply and demand dynamics. Input costs such as fertilizer and fuel significantly affect production and supply to the market; therefore, their fluctuations influence commodity prices (Ulussever et al., 2023). Other macroeconomic variables, such as exchange rates, the global price of oil, and food import costs (Abodi et al., 2021), as well as microeconomic factors such as household income (Oztornaci et al., 2024), have been cited as important predictors of domestic price movements.

In an attempt to curb oil price fluctuations, Iftikhar et al. (2025) introduced a hybrid model to predict daily crude oil prices, combining data analysis using regression, time series analysis, and machine learning methods to test the hybrid approach's ability to capture both long-term trends and short-term fluctuations. The hybrid model was applied to datasets from the Brent and West Texas Intermediate (WTI) markets, producing outstanding results. The results showed that the model produced an MAE of 1.28, an RMSE of 1.59, a Pearson's correlation coefficient of 0.94, and a Dice similarity index of 0.82, which indicates its effectiveness. Spatial and market accessibility variables, including distance from major markets and the quality of transport infrastructure, were shown to explain price variations across regions within a country (Ennaji et al., 2024). Considering the diversity of these determinants, recent literature has favored more complex models, such as XGBoost and gradient boosting, due to their proven predictive power and interpretability (Ulussever et al., 2023; Ennaji et al., 2024). Although ensemble models such as LightGBM, gradient boosting, and random forest have been applied to predict food prices, there is minimal research focused on combining XGBoost and gradient boosting in an integrated framework to achieve higher predictive accuracy. The majority of studies that emphasize commodities do not outline a generalized methodology to cover multiple types of food. Previous research indicates that hybrid forecasting models are consistently more accurate than standalone models in many fields of study, including stock markets, crude oil forecasting, financial time series, bitcoin price, and agricultural inputs such as fertilizer prices. In addition to demonstrating their effectiveness, these studies also illustrated the versatility of hybridization approaches in higher-order models, which can capture both the long-run structural components of time series data and the instantaneous stochastic characteristics. Earlier studies demonstrate a clear advantage of hybrid methods. Therefore, this study developed a hybrid XGBoost and gradient boosting model to optimize the precision of food price prediction as a valuable tool to ensure market stability and food security.

## 2 Methodology

### 2.1 Data acquisition

The data used in this study were obtained from the World Food Programme, an organization that utilizes field-based market monitoring, mobile data tools such as KoBoToolbox, and collaboration with local institutions, including the Food and Agriculture Organization (FAO), in Kenya to collect food price datasets. The organization collects this data from more than 90 countries in 1,500 markets worldwide, and it is updated weekly or monthly; Kenya is one of them. From this, Kenya's food prices data, consisting of 13 columns and 13,010 rows from January 2006 to September 2024, were extracted from their database to support this study (World Food Programme, 2025). However, the data set was supplemented with data on exchange rates in US dollars from the Central Bank of Kenya (Central Bank of Kenya, 2024).

Furthermore, data on the inflation rate International Monetary Fund (2024) were also added to help understand the effect of changes in the inflation rate on Kenyan food prices. In addition, county boundary data from OCHA's Humanitarian Data Exchange collected using GIS tools in collaboration with partners such as the United Nations Office for the Coordination of Humanitarian Affairs (UNOCHA), the World Food Program (WFP), and Kenya's Ministry of Lands were added to help in the analysis of commodity prices across different counties (Office for the Coordination of Humanitarian Affairs, 2022). This is shown in Table 1.

**Table 1:** Descriptions of the map features generated from the Kenya counties shapefile, covering the definitions of the primary parameters considered to define the geographical and spatial attributes of each county.

**Feature**	**Description**
Area	Approximate area of the county, expressed in square meters.
Perimeter	The perimeter length of the county boundary in meters.
County	The official name of the county (e.g., Nairobi).
Shape length	A representation of the county's boundary length in meters.
Shape area	An estimate of the county area in kilometers^2^.
Geometry	The actual shape of each county, with latitude and longitude coordinates used for drawing the map, measured in degrees.

### 2.2 Data pre-processing

This is an important prerequisite step in modeling that helps identify anomalies such as noise, inaccuracies, inconsistencies, and incompleteness in the dataset. This step, according to Smith and Johnson (2023), helps improve the quality of the data. Table 2 shows the main steps taken to clean up the dataset.

**Table 2:** Data cleaning operations and the associated rationale, outlining the specific pre-processing procedures executed on the dataset to increase consistency, improvement, and readiness to analyze.

**Procedure**	**Description**	**Rationale**
Feature renaming	admin1, admin2, category, and prices were renamed to region, county, commodity category, and price [in Kenyan Shillings (KES)] respectively.	This was done to ensure their clarity and consistency with the data context and to align them with their geographical representations in Kenya. Meaningful names aid in feature understanding (Mastropaolo et al., 2023).
Date formatting	Date column were converted to datetime format.	Supports feature engineering from the column and time-based analysis.
Data integration	Combined datasets from the different sources into one sheet.	Enables comprehensive analysis in multidisciplinary studies (Kathiravelu et al., 2018).

The dataset had no missing values. Extreme price values (outliers) were dealt with using the interquartile range capping and flooring method. Outliers are defined as values that fall below the lower quartile given by Equation 1 or above the upper quartile given by Equation 2. The difference between the upper and lower quartiles gives the interquartile range, which is given by Equation 3. Instead of removing these observations, we winsorized them in the upper quartile and floored them in the lower quartile. This was done to help preserve sample size and minimize the distorting effect of extreme values. This method is mainly used in classification algorithms to make them more robust (Dash et al., 2023). Similarly, simulation research comparing univariate winsorization statistics reveals improved estimator stability for interquartile range winsorization under various distributional contaminations (Abuzaid and Alkronz, 2024).


(1) $$\begin{array}{l} \text{Lower quartile}=Q_{1}-1.5\times IQR \end{array}$$

(2) $$\begin{array}{l} \text{Upper quartile}=Q_{3}+1.5\times IQR \end{array}$$

(3) $$\begin{array}{l} IQR=Q_{3}-Q_{1} \end{array}$$

### 2.3 Feature engineering

Feature engineering involves the creation of new, informative features from raw data to improve model performance and help uncover deeper insights from the data. He et al. (2024) argues that by transforming raw inputs into formats that effectively capture the underlying patterns of the problem domain, feature engineering improves both interpretability and accuracy. After converting the original date field to a Datetime format and setting it to index, the year, month, day, and day name were extracted from the column. This was necessary since these features were to help in the analysis and better understanding of the data. Additionally, unit quantity was extracted from the item unit field by splitting the item unit, which consisted of values like 10kg, into two features: unit quantity as 10 to facilitate quantitative analysis of different quantity prices, and unit measurement as kg. After splitting, the unit of the item and the unit of measurement were dropped because they were not significant and would have introduced redundant variables. These engineered features were introduced to enrich the dataset and improve the effectiveness of subsequent analysis and modeling phases.

### 2.4 Exploratory data analysis

Exploratory data analysis plays a crucial role in identifying key characteristics, examining distributions, and exploring relationships between variables. This process enables us to explore the key qualities and patterns present in the dataset in greater detail. As noted in Attobrah (2024), exploratory data analysis becomes especially significant when dealing with large datasets, as it uncovers the hidden patterns and traits that often lie just beneath the surface of the data. To make the data easier to interpret, visualizations such as stacked bar charts, vertical bar graphs, and horizontal bar graphs were employed to gather patterns, trends, and anomalies in the dataset. Statistical analysis was performed on the dataset to help understand the statistical distribution of both categorical and numerical features. Additionally, a heatmap was plotted to show the correlation between the numerical features.

### 2.5 Data transformation

To prepare the dataset for modeling, a transformation process was necessary. The first step was label encoding, which allowed us to convert categorical features such as commodity, region, price flag, and commodity category into their numerical equivalents. This is a key step because most machine learning algorithms, including the regression models we used in this study, cannot handle data in string format. As pointed out by Sharma et al. (2023), the encoding of labels assigns each category a unique integer, allowing us to incorporate these features into our model training while maintaining computational efficiency. However, it is important to note that, despite its benefits, Sharma et al. (2023) cautions that this encoding method could unintentionally create misleading ordinal relationships within nominal data. Logarithmic transformation was then performed in the price column to make commodity prices regularly distributed to reduce disparities in the numerical range of features, particularly in continuous variables, which is in line with West (2022), who found that this transformation method helps reduce skewness and stabilize variance and standard deviation (Iftikhar et al., 2024). Similarly, to assess the efficacy of early abdominopelvic computed tomography in patients with acute abdominal pain whose cause is unknown, Keene (1995) used log transformation. The skewed data was normalized, which allowed the researchers to obtain better results.

### 2.6 Feature selection and modeling

After cleaning and transforming the data, the selection of features was carried out using the Pearson correlation coefficient to select the statistically significant variables to include in the model, considering the variables with high t-statistic Equation 4) and variables whose *p*-values are below 0.05 Equation 5 also including commodity even though it had a *p*-value >0.05, but it is significant for this study.


(4) $$\begin{array}{l} t=\frac{r\cdot \sqrt{n-2}}{\sqrt{1-r^{2}}} \end{array}$$

(5) $$\begin{array}{l} p=2\cdot P\left(T_{n-2}>|t|\right), \end{array}$$

where *r* is the Pearson correlation coefficient, *n* is the number of observations, *t* is the t-statistic with *n*−2 degrees of freedom, is the *p* two-tailed p-value and *T*_*n*−2_ is the t-distribution with *n*−2 degrees of freedom.

The dataset was divided into training and test datasets of 80% and 20%, respectively. The training data was intended to help develop and train the models, while the test dataset was used to evaluate and monitor the models' performance based on the applied metrics, as explained by Dobbin and Simon (2011). The training data was then used to train the decision tree, random forest, XGBoost, gradient boosting regressors, and the hybrid model.

#### 2.6.1 Decision tree regressor

A decision tree regressor is a non-parametric supervised learning model that is used to predict a continuous output variable. It describes data by learning simple decision rules gathered from the input features. As explained by James et al. (2013), the tree splits the data into subsets based on feature values, where each internal node represents a decision on a feature, and each leaf node represents a predicted outcome.

#### 2.6.2 Random forest regressor

The random forest is a powerful ensemble learning technique that combines tree predictors. All trees in the forest depend on the values of an independently drawn random vector for each tree. Breiman (2001) explains that this model helps eliminate variance and improve predictive performance by combining the results from many decision trees. Equation 6 shows the expression for a random forest.


(6) $$\begin{array}{l} \hat{y}=\frac{1}{T}\overset{T}{\underset{t=1}{\sum }}h_{t}\left(x\right) \end{array}$$

where *ŷ* is the final prediction, *T* is the number of trees in the forest, and *h*_*t*_(*x*) is the prediction from the *t*^th^ decision tree.

#### 2.6.3 Linear regression

This is a regression model that works well with datasets in which the response variable exhibits a strong correlation, either positive or negative, with the predictor variable, indicating that changes in the predictor variable have a significant impact on the response variable (Quirk and Rhiney, 2021). It can be expressed as a simple linear regression when only one predictor variable is used, as shown in Equation 7.


(7) $$\begin{array}{l} y=\beta_{0}+\beta_{1}x+\epsilon , \end{array}$$

Where β_0_ is the intercept, β_1_ is the slope of the regression line, *x* is the independent variable, *y* is the response variable, and ϵ is the error term.

Another type of linear regression is a multiple linear regression model. In this case, the response variable is explained by several predictor variables, as shown in Equation 8 below.


(8) $$\begin{array}{l} y=\beta_{0}+\beta_{1}x_{1}+\beta_{2}x_{2}+\cdots +\beta_{p}x_{p}+\epsilon , \end{array}$$

where, *y* is the response variable, *x*_1_, *x*_2_, …, *x*_*p*_ are independent variables such as the inflation rate, the quantity of units, β_0_ is the intercept, β_1_, β_2_, …, β_*p*_ are the coefficients, and ϵ is the error term.

#### 2.6.4 XGBoost regressor

For regression tasks, the Extreme Gradient Boosting Regressor is a popular and effective ensemble learning method. It corrects the residuals of earlier decision trees by training them sequentially to build a model. To control model complexity, the model is optimized by minimizing a loss function with an additional regularization term that regulates complexity and prevents overfitting (Chen and Guestrin, 2016). The loss function for XGBoost in predicting food prices is provided by Equation 9.


(9) $$\begin{array}{l} L\left(\theta \right)=\overset{n}{\underset{i=1}{\sum }}{\left(P_{i}-{\hat{P}}_{i}\right)}^{2}+\overset{K}{\underset{k=1}{\sum }}\Omega \left(f_{k}\right), \end{array}$$

where *P*_*i*_ is the actual market price of a food commodity for observation *i*, ${\hat{P}}_{i}$ is the predicted price based on the output of the model, *X*_*i*_ is the feature vector for observation *i* (including the quantity per unit, the exchange rate of the currency, the type of commodity, and so on) and the regularization term Ω(*f*_*k*_) is as shown in Equation 10.


(10) $$\begin{array}{l} \Omega \left(f_{k}\right)=\gamma T_{k}+\frac{1}{2}\lambda \overset{T_{k}}{\underset{j=1}{\sum }}w_{k,j}^{2}, \end{array}$$

where the weight of the *j*-th leaf in this tree is represented by *w*_*k, j*_, while *T*_*k*_ is the number of leaves in the *k*-th tree. For excellent accuracy and generalization performance. Chen and Guestrin (2016) explains that the XGBoost regressor optimizes a regularized loss function using an additive ensemble of decision trees.

#### 2.6.5 LightGBM

Light Gradient Boosting Machine (LightGBM) is an efficient gradient boosting framework based on decision trees. LightGBM builds an ensemble of weak learners (decision trees) in a sequential manner, where each new tree is trained to minimize the residuals of the previous trees. It distinguishes itself from other boosting frameworks by adopting a leaf-wise tree growth strategy, which allows it to achieve a lower loss compared to level-wise growth strategies, especially in large datasets (Ke et al., 2017).

The loss function optimized by LightGBM for regression is defined as follows:


(11) $$\begin{array}{l} L\left(\theta \right)=\overset{n}{\underset{i=1}{\sum }}\ell \left(P_{i},{\hat{P}}_{i}\right)+\overset{K}{\underset{k=1}{\sum }}\Omega \left(f_{k}\right), \end{array}$$

where *P*_*i*_ is the actual market price of a food commodity for observation *i*, ${\hat{P}}_{i}$ is the predicted price of the model, ℓ(·) is a differentiable convex loss function and Ω(*f*_*k*_) is the regularization term that penalizes the complexity of the *k*-th regression tree.

The regularization term is given by the following equation:


(12) $$\begin{array}{l} \Omega \left(f_{k}\right)=\gamma T_{k}+\frac{1}{2}\lambda \overset{T_{k}}{\underset{j=1}{\sum }}w_{k,j}^{2}, \end{array}$$

where *T*_*k*_ is the number of leaves in the *k*-th tree, and *w*_*k, j*_ represents the weight of the *j*-th leaf. Using histogram-based continuous variable segmentation and a leaf-wise split approach, LightGBM achieves high efficiency and scalability, making it particularly suitable for large-scale food price prediction tasks that involve high-dimensional economic and commodity data (Ke et al., 2017).

#### 2.6.6 Gradient boosting regressor

This is a robust machine learning algorithm. It uses gradient descent to minimize the log loss function, constructing an additive model step by step. In addition, Friedman (2001) states that the model is ideally suited to operate with imperfect data, since it produces competitive and very stable algorithms for regression and classification problems.

#### 2.6.7 Hybrid model

After evaluating the performance of individual regression models, we employed the ensemble stacking technique to construct a hybrid model, aiming to achieve greater accuracy and robustness. We used XGBoost and gradient boosting as base models, as these models can learn complex nonlinear relationships and reduce residual errors via iterative boosting methods. The models were trained separately on the training set, and their outputs served as input features for the next stage of modeling. A linear regression model was then used as a meta-learner to synthesize the results of the base models into one prediction. The motivating factor for employing linear regression as a meta-learner was its simplicity and interpretability because it can provide an error-minimizing combination of the predictions of the base learners. The two-level training framework enabled the hybrid model to fully utilize the complementary benefits of XGBoost regularization and efficiency alongside the adaptability of gradient boosting in learning residuals while reducing the chances of overfitting. This approach has been applied in the prediction of fresh agricultural products by Zhang et al. (2025), who proposed a lightweight gradient boosting model to enhance prediction accuracy.

### 2.7 Evaluation

The models were then evaluated to select the most suitable one for implementing in the prediction of food prices. In regression modeling, evaluation metrics help quantify the performance of predictive models. The metrics help determine the accuracy, magnitude of error, and overall reliability of the predictions. The next section explains the primary evaluation metrics, along with their corresponding mathematical formulations and explanations, which were used to illustrate the suitability of the models.

#### 2.7.1 Mean squared error

The mean squared error (MSE) is a metric used to evaluate regression models, measuring the average of the squared differences between actual and predicted values. Equation 13 shows the mathematical representation of the metric.


(13) $$\begin{array}{l} MeanSquaredError=\frac{1}{n}\overset{n}{\underset{i=1}{\sum }}{\left(y_{i}-{\hat{y}}_{i}\right)}^{2}, \end{array}$$

where *n* is the number of observations, *y*_*i*_ is the actual price, and ŷ_*i*_ is the predicted price.

The metric penalizes bigger errors more than smaller ones, making it sensitive to outliers. The lower the MSE, the better the model, so a smaller mean square error is preferred.

#### 2.7.2 Root mean squared error

This is an evaluation metric that measures the average magnitude of prediction errors. Here, the errors are squared before averaging and then taking the square root, as illustrated in Equation 14. It can also be obtained manually by taking the square root of the mean squared error.


(14) $$\begin{array}{l} RootMeanSquaredError=\sqrt{\frac{1}{n}\overset{n}{\underset{i=1}{\sum }}{\left(y_{i}-{\hat{y}}_{i}\right)}^{2}} \end{array}$$

It represents the error magnitude in the same units as the target variable, making it interpretable for real-world applications.

#### 2.7.3 Mean absolute error

This metric measures the average absolute difference between actual values and predicted values. The metric result indicates the distance between the predictions and the actual values. Equation 15 shows its mathematical representation.


(15) $$\begin{array}{l} MeanAbsoluteError=\frac{1}{n}\overset{n}{\underset{i=1}{\sum }}|y_{i}-{\hat{y}}_{i}| \end{array}$$

It is less sensitive to outliers compared to the mean squared error.

#### 2.7.4 R-squared score

This is a regression evaluation metric that indicates the proportion of variance in the response variable that can be predicted from the predictor variables. Equation 16 below shows a mathematical expression. This metric is commonly used to show how well the model fits the data.


(16) $$\begin{array}{l} R^{2}=1-\frac{\overset{n}{\underset{i=1}{\sum }}{\left(y_{i}-{\hat{y}}_{i}\right)}^{2}}{\overset{n}{\underset{i=1}{\sum }}{\left(y_{i}-ȳ\right)}^{2}}, \end{array}$$

where ȳ is the mean of actual prices. It measures the proportion of variance in the response variable that the model explains, ranging from 0 to 1.

### 2.8 Diebold-Mariano test

After evaluating the models, we implemented the Diebold-Mariano test to statistically analyze how the accuracy in predictions of the models differed significantly from each other. This is a standard approach used to assess the predictability of competing models (Diebold and Mariano, 1995). In particular, the Diebold-Mariano test's null hypothesis assumes that the competing models have equal predictive accuracy, while the alternative hypothesis posits that one model has superior predictive accuracy compared to the other.

First, the loss differential of the compared models is computed. If, at time *t*, *e*_1, *t*_ and *e*_2, *t*_ are the predictive errors of models 1 and 2, respectively, and *g*(·) is a loss function, then the loss differential can be written as in Equation 17:


(17) $$\begin{array}{l} d_{t}=g\left(e_{1,t}\right)-g\left(e_{2,t}\right), \end{array}$$

where *d*_*t*_ represents the difference in loss between the two models at observation *t*.

The mean loss differential across *T* predictions is then computed using Equation 18:


(18) $$\begin{array}{l} \bar{d}=\frac{1}{T}\overset{T}{\underset{t=1}{\sum }}d_{t} \end{array}$$

To determine whether the mean difference is significantly different from zero, we calculate the DM statistic using Equation 19:


(19) $$\begin{array}{l} DM=\frac{\bar{d}}{\sqrt{\frac{{\hat{\gamma }}_{0}+2\overset{h-1}{\underset{k=1}{\sum }}{\hat{\gamma }}_{k}}{T}}}, \end{array}$$

where ${\hat{\gamma }}_{k}$ is the estimated autocovariance of the loss differential at lag *k*, *h* is the prediction horizon, and *T* is the number of predictions.

Due to its asymptotic properties, the *DM* statistic can be treated as a standard normal statistic, facilitating statistical inference. Suppose that the test statistic is statistically significantly different from zero. In that case, it means that we reject the null hypothesis of equal predictive accuracy, which, in turn, means that one model provides significantly better forecasts than the other.

### 2.9 Model validation

The regression models developed were subjected to a validation procedure, and the performance and generalizability of the models were assessed. Both the standalone regression models and the hybrid model underwent analysis stages. Each model was trained on the log-transformed target variable separately and then evaluated using a 5-fold cross-validation to assess its stability and robustness. For model validation purposes, the R-squared score was used as the criterion measure, averaged across folds, describing how much variance in the data is explained by each model. The procedure follows current best practices to prevent overfitting and yields a more accurate estimate of performance on unseen data. This is interpreted in Equation 20.


(20) $$\begin{array}{l} {\bar{R}}^{2}=\frac{1}{k}\overset{k}{\underset{i=1}{\sum }}R_{i}^{2}, \end{array}$$

where *k* is the number of folds, $R_{i}^{2}$ is the R-squared score for the *i*^th^ fold, and ${\bar{R}}^{2}$ is the average R-squared score across all folds.

### 2.10 Optimization

To optimize the model's performance, a randomized search was applied during the tuning process. For example, Probst et al. (2019) emphasized that parameterization can significantly influence the performance of ensemble models such as random forests and XGBoost. Similarly, Ali et al. (2023) demonstrated that random search and Bayesian optimization outperform manual and grid search methods in multidimensional spaces, which, in turn, lead to improved model performance.

### 2.11 Feature importance

Analyzing the importance of features was necessary to identify which features have the most significant impact on predicting the response variable. This process helps to understand and capture the underlying relationships in the data, thereby optimizing model performance. As supported by Pudjihartono et al. (2022), feature selection not only improves model prediction accuracy but also reduces computational costs by including only the most relevant features, thereby enhancing efficiency and effectiveness in deployment. To help strengthen the importance of the feature, an interpretability analysis on the hybrid model was conducted using SHAP. The SHAP values for the hybrid model were calculated as the mean of the SHAP outputs from its base learners. A beeswarm summary plot was created to visualize the important features. In addition, to help check for bias and the stability of the model's prediction across different commodity categories, such as cereals, we conducted a residual analysis. The residuals were computed by subtracting the actual value from the predicted value in the test data. For each commodity category, the mean residual, standard deviation, minimum, and maximum residuals were summarized to catch tendencies of underestimation or overestimation and variance in error. This approach helps to identify systematic biases that other error metrics often overlook (Verma, 2025), highlighting the model's shortcomings in predicting specific commodities.

### 2.12 Deployment

To make the results of the model available to farmers, stakeholders, policymakers, and other users, a web application was developed using the Django framework (Django Software Foundation, 2025). The deployment included building a simple interface on the front end. This interface features user registration, authentication, and a prediction form that is dynamically generated based on the ten most important features chosen during model training. In the back end, the pre-trained hybrid machine learning model was integrated, along with the relevant feature encoder. This setup processes incoming user inputs, applies the needed transformations, and provides predictions to the users. The web application codes are stored in my GitHub repository for use and further improvement (benard3360-star, 2025).

## 3 Results

### 3.1 Data pre-processing and feature engineering

**Figure 1:**
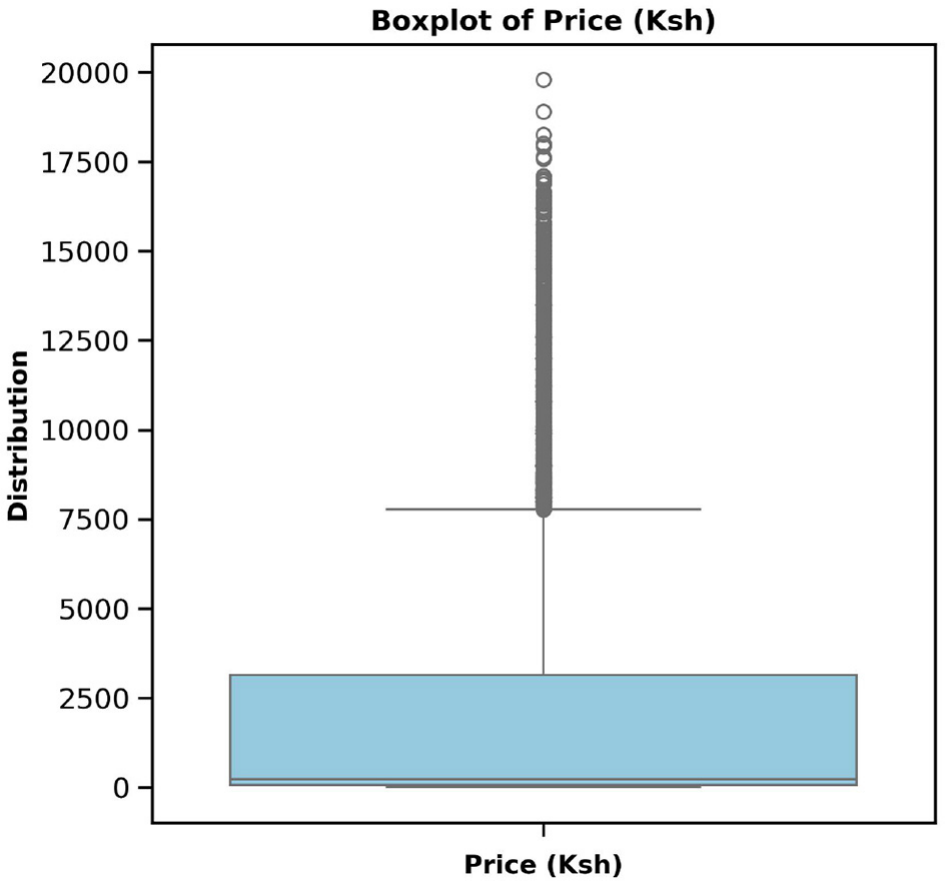
Commodity price boxplot before dealing with the outliers. The plot shows that the prices go up to KES. 19,800, which is far above the upper whisker, thus necessitates addressing these large values that may hinder model performance.

**Figure 2:**
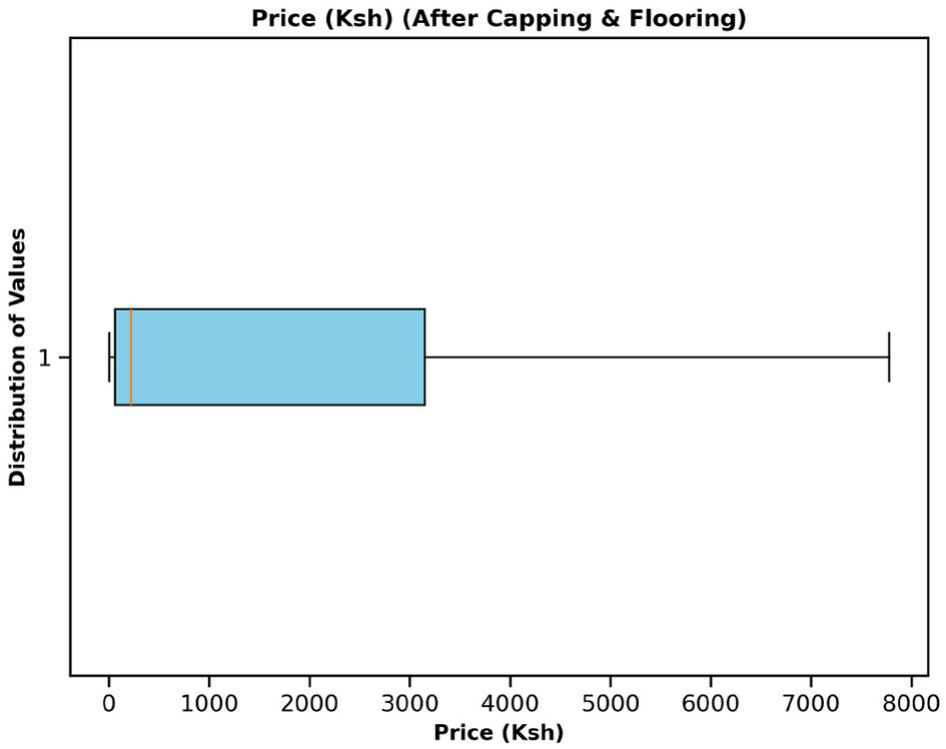
Commodity price boxplot after applying capping and flooring to deal with the outliers.

**Figure 3:**
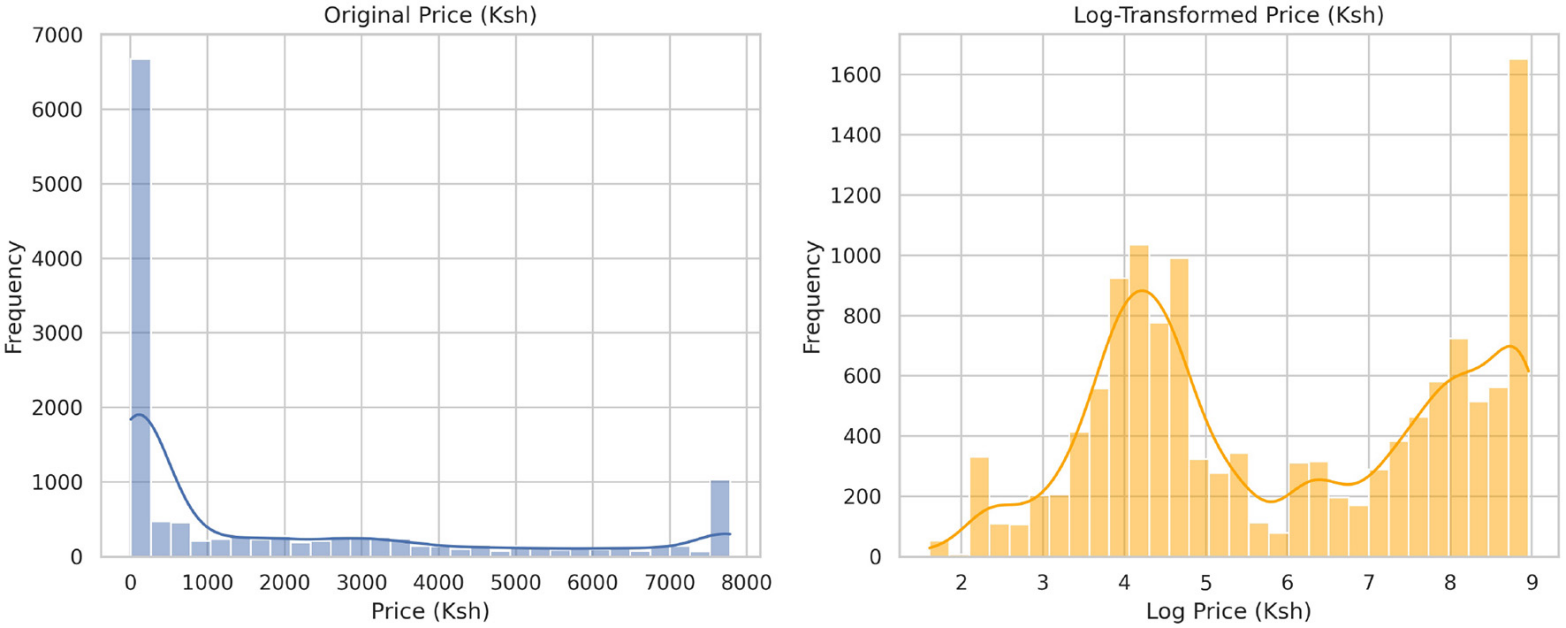
Figure showing the original distribution of prices before transformation and after the log transformation. From this, the distribution appears to be bimodal after the transformation.

Table 3 presents the preprocessed data features, revealing that all features are complete with no null values and that all features are consistent. The final data have been combined with records from various sources, as explained in Section 2.1. This integration allowed the construction of a multidimensional dataset to support complex modeling and advanced analysis at various levels. The interquartile range was then applied to the price column to identify the outliers, to which capping and flooring were applied to deal with them, as illustrated in Figures 1, 2. The free price variable of the outlier was then logarithmically transformed to reduce the skewness and variability, as in Table 4, showing that the price column is skewed with a kurtosis of 3.59, which is above the range of (–2,+2), thus in need of transformation. The log transformation effectively reduced skewness and stabilized the variance, producing a distribution that is more suitable for modeling, as illustrated in Figure 3.

**Table 3:** A description of all the variables contained in the dataset used for exploratory data analysis and modeling.

**Feature**	**Count**	**Data type**	**Description**
Region	13,010	object	Geographical region where the data was collected
County	13,010	object	Specific county within the region
Market	13,010	object	Market name where the commodity price was recorded
Commodity category	13,010	object	Classification of the commodity (e.g., cereals and tubers)
Commodity	13,010	object	Specific name of the commodity (e.g., maize, beans)
Price flag	13,010	object	Flag indicating whether the price is aggregated or actual
Price type	13,010	object	Type of price, such as wholesale or retail
Currency	13,010	float64	Exchange rate of Kenyan Shillings to USD
Price	13,010	float64	Price of the commodity quantity in Kenyan shillings
Inflation rate	13,010	float64	General inflation rate at the time of pricing
Unit quantity	13,010	int64	Quantity of the item being priced (e.g., per kg, per liter)
Month	13,010	int32	Month of the year when the data was collected
Day name	13,010	object	Day of the week (e.g., Monday, Tuesday)
Day classification	13,010	object	Classification of the day (e.g., weekday or weekend)
Log price	13,010	float64	Log-transformed price in Kenyan shillings

**Table 4:** Descriptive statistics for numerical variables. According to the table, the highest currency amount is 160 dollars, and the highest commodity price is 19,800 shillings.

**Variable**	**Mean**	**Standard deviation**	**Median**	**Minimum value**	**Maximum value**	**Kurtosis**
Currency	108.62	19.84	109.26	40.38	160.24	–0.5118
Price	2,093.12	3,182.10	220.00	5.00	19,800.00	3.5859
Inflation rate	6.83	2.74	6.32	1.85	19.72	–0.3815
Unit quantity	61.91	106.56	13.00	1.00	500.00	9.4100
Month	6.07	3.40	6.00	1.00	12.00	–1.1641

### 3.2 Exploratory data analysis

**Figure 4:**
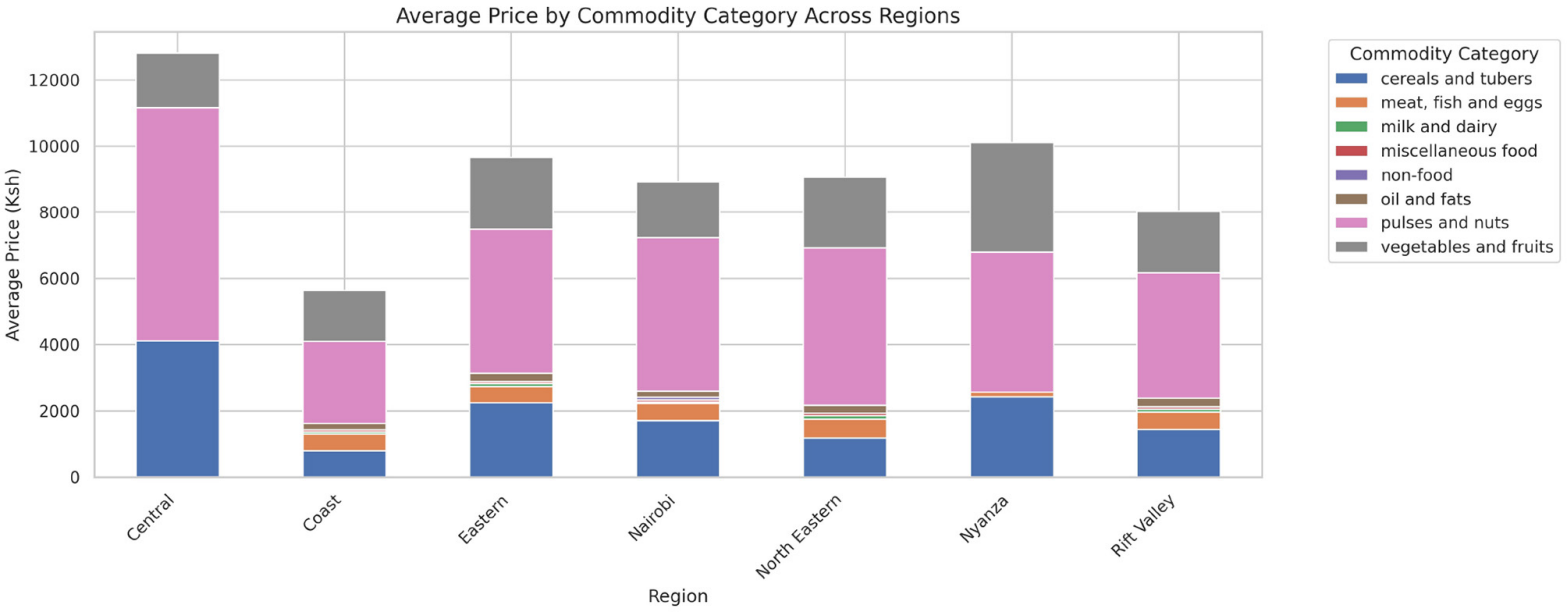
Average prices of commodities by category across different regions. Pulses and nuts appear to be the food commodities with the highest average prices across all regions.

**Figure 5:**
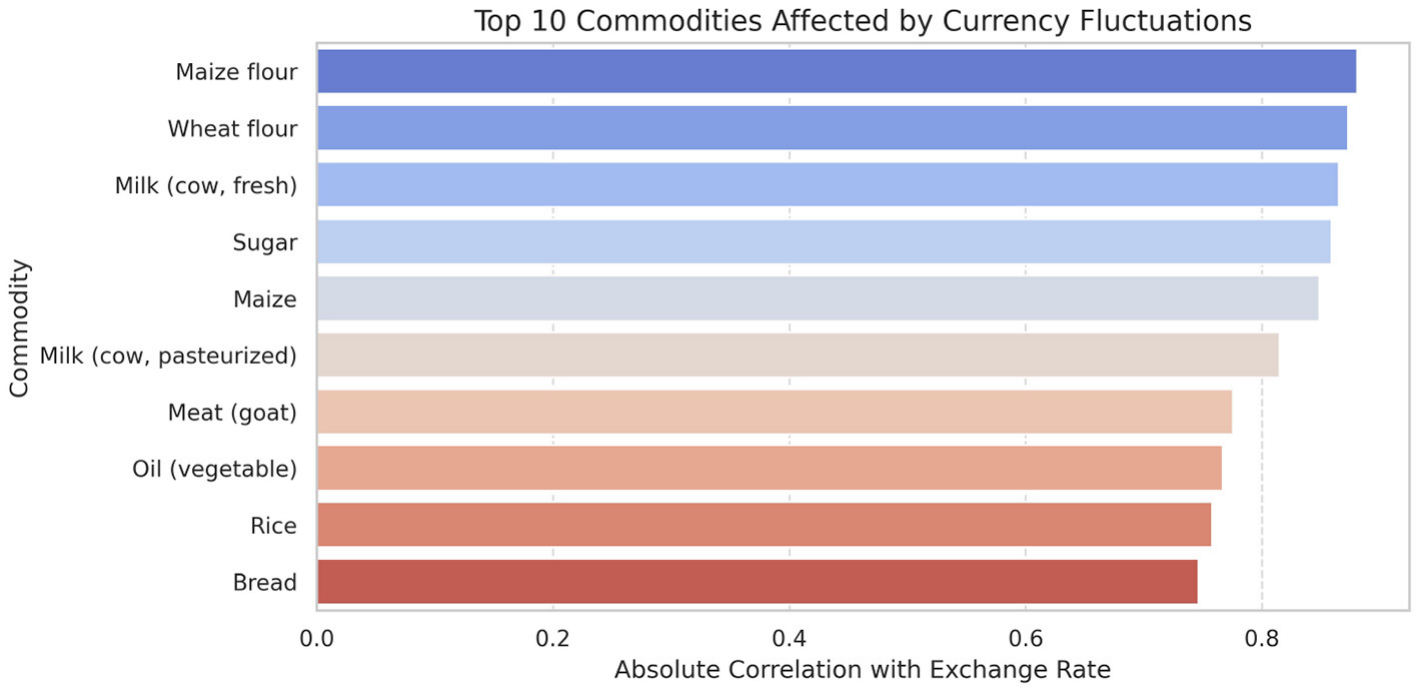
Currency (USD) effect on commodities. Maize flour appears to be highly affected by the fluctuations in the currency.

**Figure 6:**
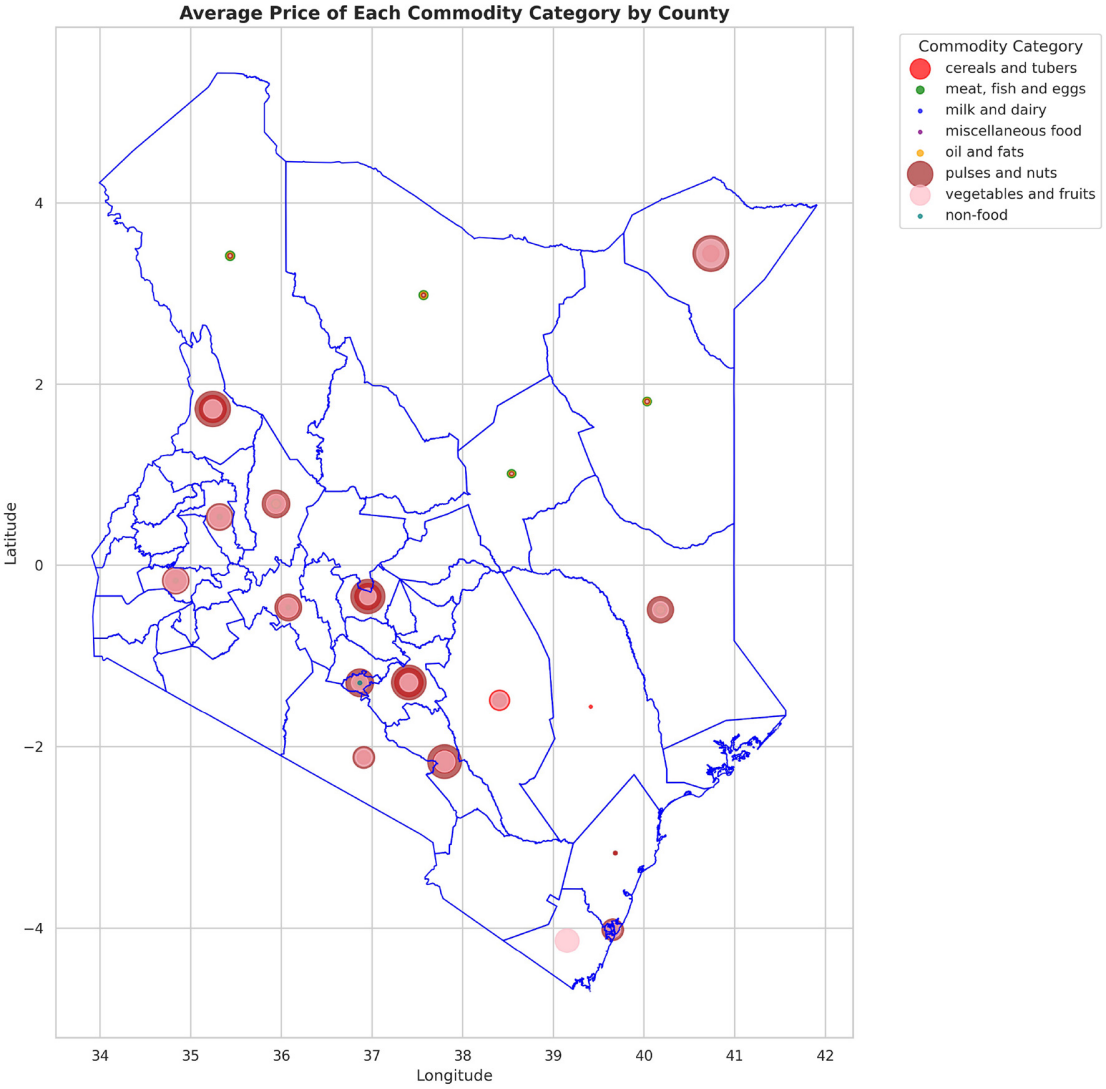
Variation in the average price of different types of commodities across counties, highlighting how geographic location influences commodity pricing trends.

**Figure 7:**
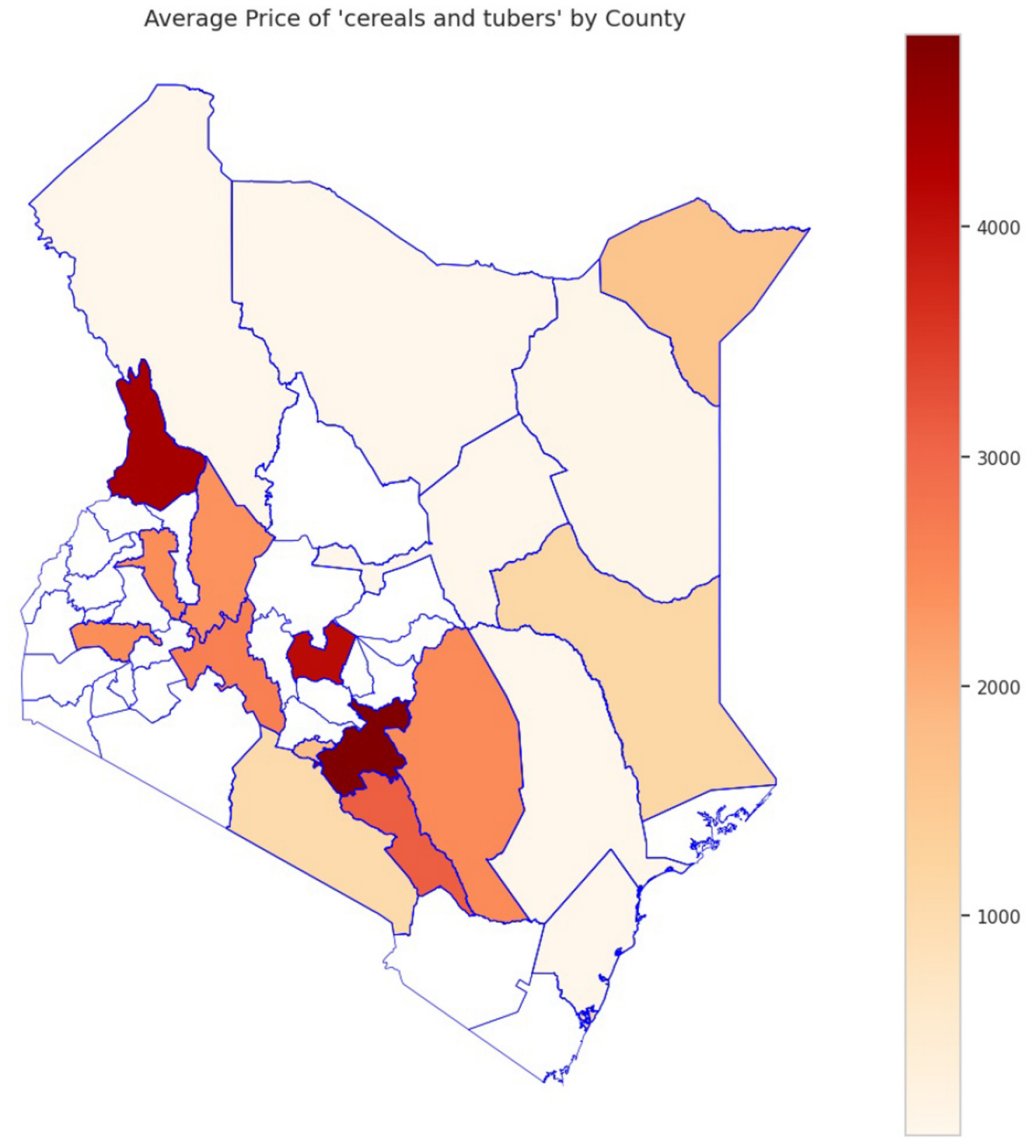
Sample analysis of variation in the average price of cereals and tubers across counties, with the dark areas indicating counties with higher average prices.

**Figure 8:**
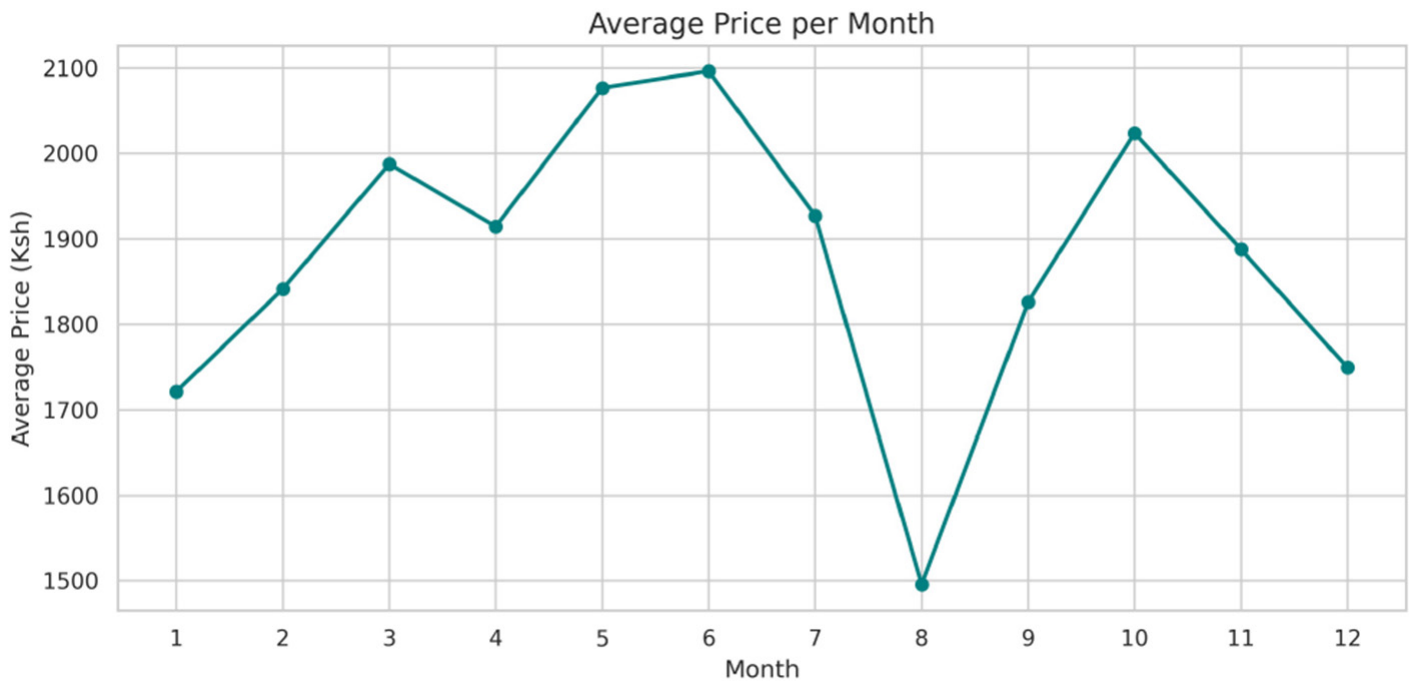
Average price of commodities per month.

The analysis presented in Table 5 reveals that the markets surveyed comprised 59 locations in 22 counties, covering eight different commodity categories, which resulted in the documentation of 47 distinct commodities. Looking at the numerical data in Table 4, currency values fluctuate between 40.38 and 160.24 dollars over various years. The lowest price for any commodity is 5 shillings, while some can go as high as 19,800.00 shillings. The results in Figure 4 show that pulses and nuts stand out as the categories of commodities with the highest average price across the regions, followed by cereals and tubers. In addition, Figure 5 is a visualization of the top ten commodities whose price values are primarily influenced by fluctuations in currency values, highlighting the economic impact of exchange rate fluctuations on our local markets. Furthermore, analysis revealed that there are dominant spatial, temporal, and categorical variations in the prices of goods throughout Kenya, as shown in Figures 6–8, illustrating that there are dominant spatial, temporal, and categorical variations in the prices of goods in Kenyan counties, illustrating the complicated relationship between agricultural production and economic activities.

**Table 5:** Summary statistics for categorical variables. Eight commodity categories were surveyed across 59 markets in 22 counties in Kenya.

**Variable**	**Unique**	**Top**	**Freq**
Region	7	Rift Valley	5221
County	22	Nairobi	2701
Market	59	Nairobi	1295
Commodity category	8	Cereals and tubers	5926
Commodity	47	Beans	1136
Price flag	2	Actual	8224
Price type	2	Wholesale	6819

### 3.3 Feature selection, modeling evaluation and optimization

**Figure 9:**
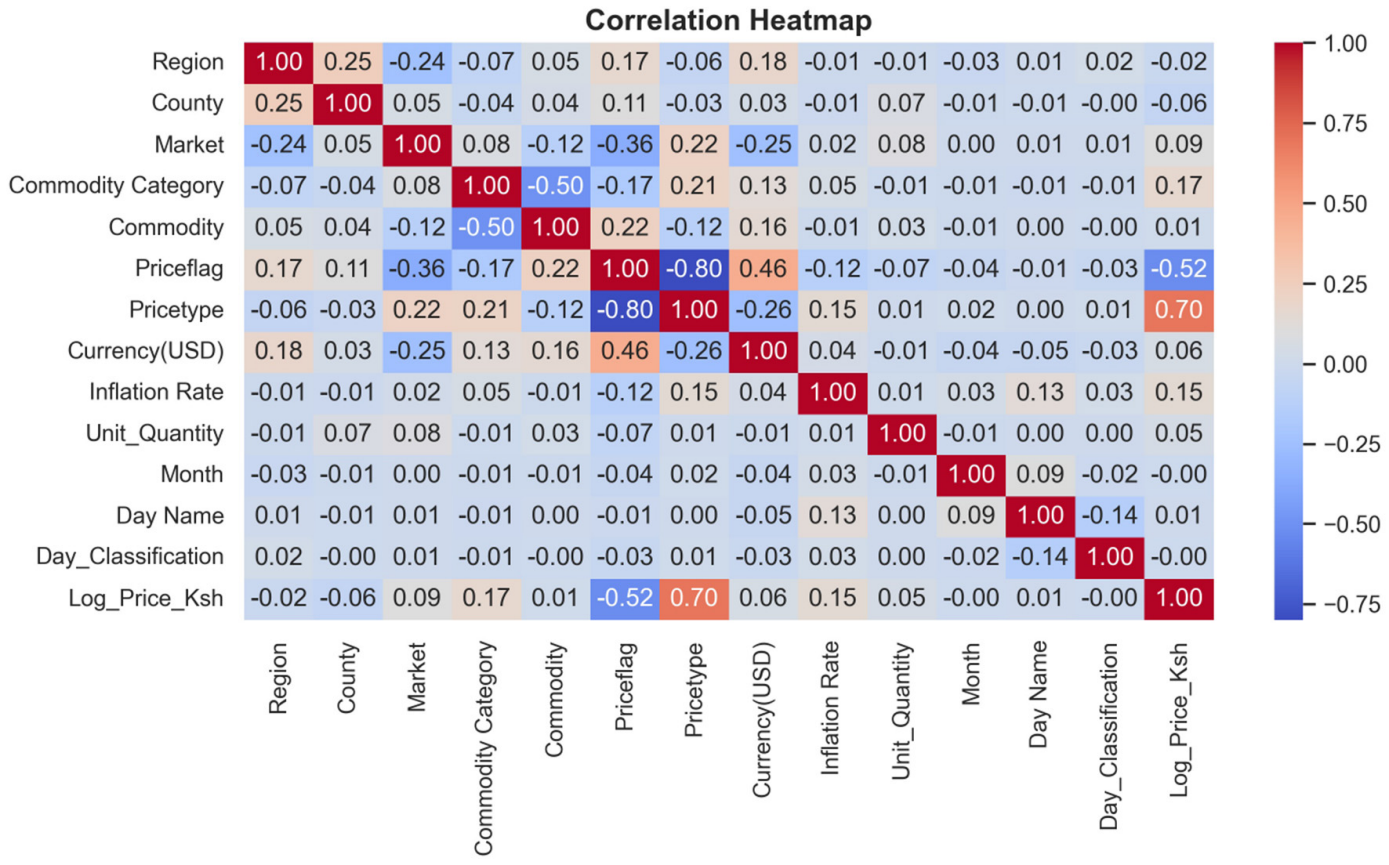
A heatmap showing how different features are related. This is the overall correlation heatmap, which shows how different features may impact the model if they are included in the model generation process.

The transformed price variable, log-price-KES, was used as the dependent variable, and the optimal features in Table 6 were used as predictors due to their statistical significance. Figure 9 presents the correlation matrix, which highlights the strength of linear associations among the features using a heatmap. The correlation heatmap illustrates the correlation between the label-encoded categorical variables and the numeric predictor variables, as well as their correlation with the response variable. From these, regression models were fitted on the training set and then evaluated by testing their predictive powers on the testing set. The overall accuracy of the standalone and hybrid models, after optimization, was as follows: the hybrid model achieved a higher R^2^ of 0.9940 and a lower mean squared error of 0.0261. The hybrid model outperformed individual models and achieved the lowest error metrics, with an R^2^ of up to 0.9940 after fine-tuning. The random forest and LightGBM also performed well; however, their residual errors and R^2^ values were inferior, indicating that the hybrid model has an edge due to its combined efforts in distinct boosting dynamics and the overall performance outcome. In contrast, linear regression did not effectively model nonlinearities and residuals, as indicated by high errors and low explanatory power, R^2^ = 0.5542, as presented in Table 7.

**Table 6:** Correlation coefficients, *p*-values, t-statistics, skewness, and kurtosis for selected variables to be used as predictor variables.

**Variable**	**Correlation coefficient**	***p*-value**	**T-statistic**	**Skewness**	**Kurtosis**
Pricetype	0.6965	0.0000	110.7140	–0.0967	–1.9907
Commodity category	0.1685	0.0000	19.4915	0.3466	–1.6734
Inflation rate	0.1489	0.0000	17.1751	0.3770	–0.3815
Market	0.0930	0.0000	10.6553	0.0357	–1.3019
Currency	0.0551	0.0000	6.2910	–0.0312	–0.5118
Unit quantity	0.0463	0.0000	5.2863	3.0260	9.4060
Commodity	0.0085	0.3310	0.9722	–0.0326	–1.1921
Region	–0.0210	0.0167	–2.3925	–0.4181	–1.1146
County	–0.0556	0.0000	–6.3522	–0.6855	–0.8050
Priceflag	–0.5237	0.0000	–70.1175	0.5480	–1.6997

**Table 7:** A table showing the comparison of model performance metrics. The results show that tree-based models such as XGBoost, random forest, and LightGBM perform well, whereas linear regression performs poorly.

**Model**	**MAE**	**MSE**	**RMSE**	**R^2^ Score**
XGBoost	0.1149	0.0342	0.1849	0.9924
Random forest	0.1162	0.0400	0.2000	0.9923
LightGBM	0.1315	0.0358	0.1891	0.9918
Decision tree	0.1328	0.0565	0.2377	0.9320
Gradient Boost	0.1865	0.0729	0.2700	0.9930
Linear regression	1.0593	1.9516	1.3970	0.5542
Hybrid model	0.1152	0.0290	0.1702	0.9934
Fine-Tuned Hybrid model	**0.1050**	**0.0261**	**0.1615**	**0.9940**

### 3.4 Model validation

Table 8 presents the performance scores obtained using a 5-fold cross-validation strategy applied to all tested models, both standalone and hybrid. The table contains the R^2^ scores for the training and test data sets, as well as the mean cross-validated R^2^ and the corresponding standard deviation. This constant validation method guaranteed dependability and comparability of the models' performances. In Table 9, we look at how residuals are distributed in different commodity categories. The majority of categories with mean residuals close to zero indicate that the predictions are error-neutral. However, some categories exhibit more pronounced skewness. For example, category 2, which contains wheat and cooking oil, exhibits the most variability with a standard deviation of 0.2061, a maximum of 1.0449, and a minimum of –0.6878. This means that the model tends to underestimate the higher prices. Category 7 also exhibits high variance, but in addition to positive and negative skew, it fails to capture significant price volatility in either direction (max = 0.9347, min = –1.0579). Category 1, in contrast, is more monotonically priced, resulting in more stable predictions, as evidenced by the low standard deviation (0.0764) and the small maximum and minimum residuals. These results suggest that the hybrid model is highly powerful and accurate in its predictions, but struggles to capture seasonality in some commodities due to the limited data available.

**Table 8:** A table showing the comparison of model R^2^ validation metrics from the applied cross-validation.

**Model**	**Train (R^2^)**	**Test (R^2^)**	**Cross-validated (R^2^)**	**Standard deviation**
Random forest	0.9990	0.9923	0.9290	0.0038
XGBoost	0.9971	0.9924	0.9926	0.0012
LightGBM	0.9967	0.9918	0.9925	0.0008
Gradient boosting	0.9843	0.9930	0.9928	0.0014
Decision tree	0.9964	0.9320	0.9893	0.0008
Linear regresion	0.5571	0.5542	0.5557	0.0408
Hybrid model	0.9934	0.9940	0.9931	0.0011

The table shows that, for example, the random forest tends to overfit.

**Table 9:** Residual analysis summary across different commodity categories. This helps identify tendencies to underestimate or overestimate and shows the spread of errors across different commodity categories.

**Commodity category**	**Mean residual**	**Standard deviation**	**Maximum residual**	**Minimum residual**
0	0.0016	0.1616	0.9267	–0.8256
1	–0.0043	0.0764	0.2474	–0.3246
2	0.0303	0.2061	1.0449	–0.6878
3	–0.0123	0.0946	0.3662	–0.4548
4	–0.0434	0.1982	0.2629	–0.6121
5	–0.0060	0.1066	0.3832	–0.4290
6	0.0039	0.0985	0.5114	–0.5267
7	–0.0049	0.2613	0.9347	–1.0579

The Diebold-Mariano test, synthesized from various metrics, illustrates that the hybrid model distinctly surpasses decision tree (DM = −7.14, *p* = 0.0000), gradient boosting (DM = −16.62, *p* = 0.00000), and LightGBM (DM = −7.18, *p* = 0.0000). The hybrid model persistently demonstrates a lower prediction error with the squared loss function. Furthermore, the results of the DM test complement the statements made previously, analyzing the mean absolute error and RMSE metrics, and state, with high confidence, that the hybrid model is more accurate and precise in prediction compared to the standalone models, as illustrated in Table 10.

**Table 10:** Results of the Diebold-Mariano test comparing the hybrid model to other standalone models.

**Compared model**	**DM_statistic**	**p_value**	**Significance**
LightGBM	–7.181494	0.0000	Hybrid better
Gradient boosting	–16.619090	0.0000	Hybrid better
Decision tree	–7.141957	0.0000	Hybrid better

Negative DM statistics with very small p-values indicate that the hybrid model performs significantly better.

### 3.5 Feature importance

**Figure 10:**
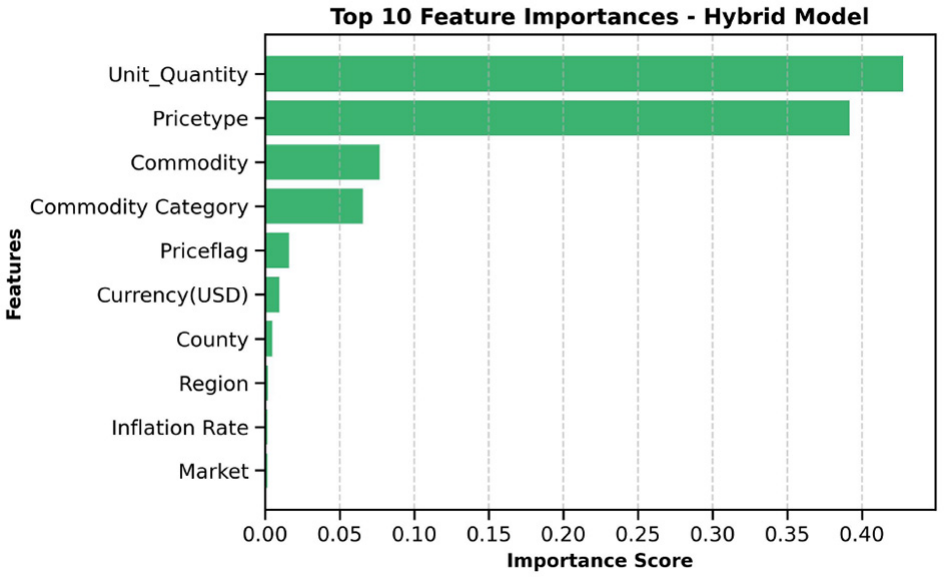
Important features influencing food price. Unit quantity appears to be the most important feature in predicting food prices.

**Figure 11:**
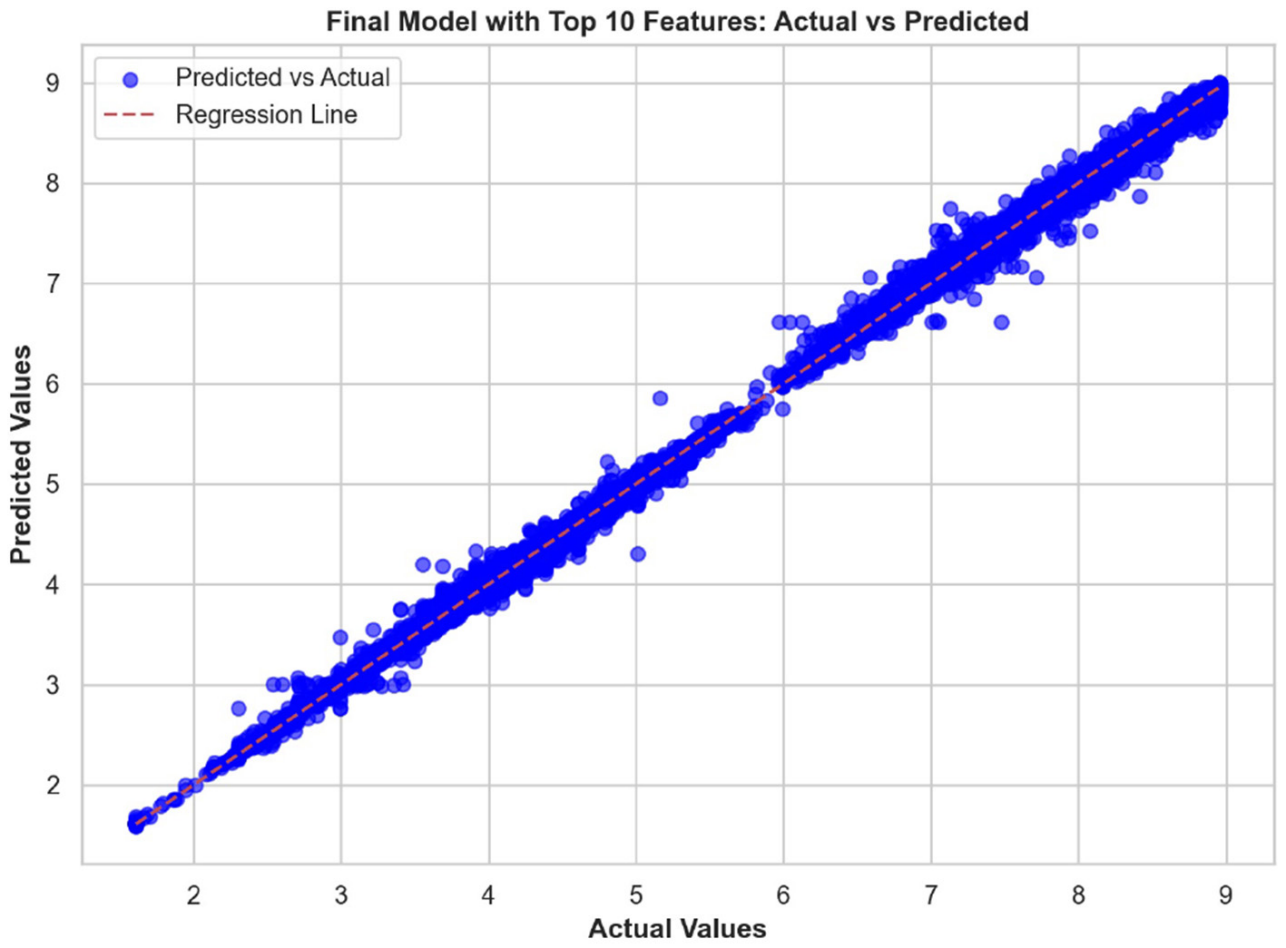
SHapley Additive exPlanations (SHAP) summary plot illustrating the relative importance and impact of key features on food price prediction using the hybrid model.

**Figure 12:**
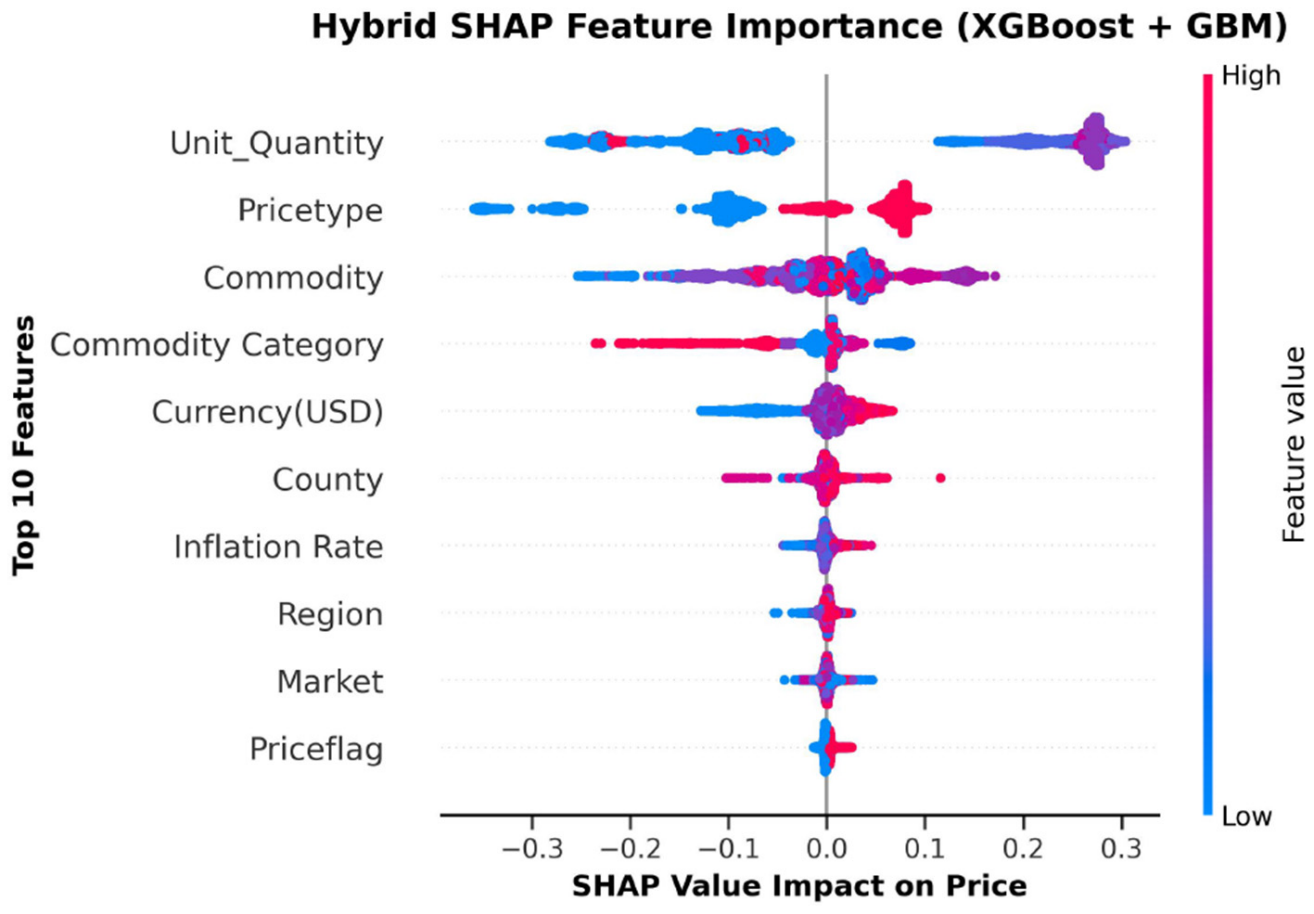
Actual commodity price compared to predicted price. The graph shows a strong linear relationship between them, indicating the effectiveness of our model in making predictions.

The top ten most important features from the hybrid model are illustrated in Figures 10, 11. These visualizations summarize the feature rankings derived from the model, as well as those ranked using SHAP, revealing that unit quantity and price type are the most intuitive features in food price prediction. Furthermore, Figure 12 shows a linear plot between actual and predicted food prices using the top 10 selected features. The plot also includes a line of best fit for the predictions, providing an indication of the accuracy of the predictions. The visualization indicates that the model tends to learn more effectively on the test data, resulting in minimal errors.

### 3.6 Deployment

**Figure 13:**
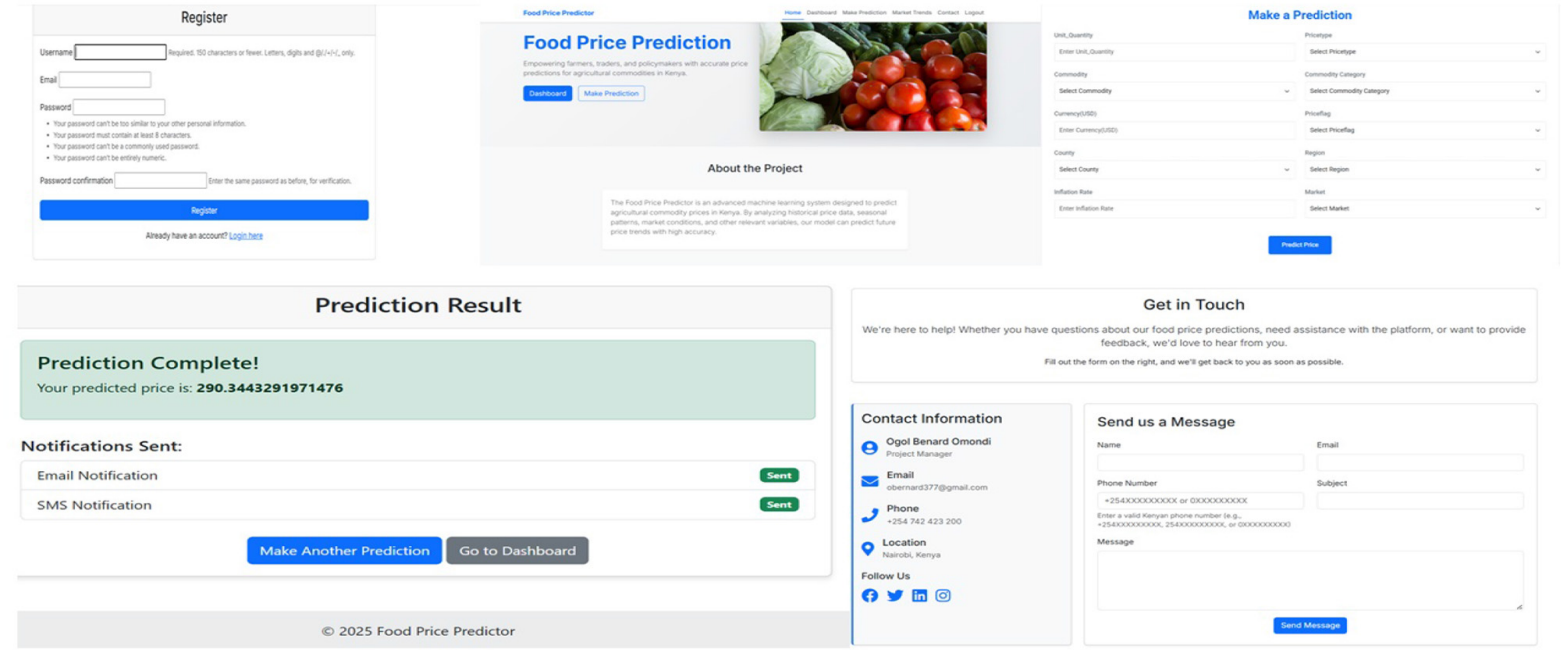
Deployed web application interface with all four compartments shown. The signup or login page, prediction page, email delivery for the predicted price, and the contact page where users can reach the website owner.

The architecture of the deployed prediction system, comprising four loosely coupled components, is presented in Figure 13: a registration and login user interface, a feature-based input form, a backend prediction engine, and an email delivery mechanism. The input form is dynamically generated based on the top ten features considered during model development. The trained hybrid model is loaded, along with the encoder and a list of feature importance, to enable uniform pre-processing during runtime. The frontend uses Django; meanwhile, the backend was implemented in Python using the Django framework. After going through input validation, the prediction form is forwarded to the encoder, and the model is saved.

## 4 Discussion

This study focused on predicting food prices in Kenya using a hybrid machine-learning model. The study's findings demonstrate that data pre-processing enhances data quality, consistency, and accuracy, thereby making the data suitable for analysis and modeling. This finding follows Maharana et al. (2022), who argued that ensuring data quality is crucial for achieving better results in supervised machine learning. Multidimensional data analysis was made possible by combining data from different organizations, which went beyond basic price prediction to consider the effects of macroeconomic variables. This integration from several sources opened up a broader perspective, similar to the multidisciplinary approach promoted by Kathiravelu et al. (2018). Through the reconciliation of variables such as the inflation rate, exchange rate, and market price from different markets, the dataset became a new canvas for testing model assumptions. This was essential not only for capturing the seasonal behavior of prices but also for the impact of structural and policy factors on food prices. As a prominent example, feature engineering was a major source of dataset expansion, particularly in terms of extracted numerical and date attributes. The newly formed features paved the way for capturing temporal dependencies and contextual aspects that the initial data alone could not provide. Their presence increased the data richness and significantly improved the model's performance in the prediction tasks. Heaton (2016) laments that feature engineering helps increase the number of features or replace existing ones to uncover underlying insights from the data, which differs for different models used. In other words, pre-processing and feature engineering not only enabled the training of stable models but also became a means of realizing the mechanisms underlying the dynamic behavior of food prices. In addition, log transformation was done to the price column, which helped to reduce the skewness of the price distribution, stabilized the variance over time, and lessened the effect of extreme price outliers (Iftikhar et al., 2024), which often occur in food commodity markets due to shocks or seasonal shortages, which for this dataset timing might have been as a result of 2020 COVID-19, which resulted in unexpected disruption in agricultural food supply chains with food prices escalating. Overall, this impacted food security as well as urban livelihoods for many households that rely on urban markets to fulfill their food expenditures (Kunyanga et al., 2023). Additionally, tree-based models such as XGBoost and gradient boosting may become unstable due to the extreme right skewness and notable outliers in the original price variable. The transformation produced a more balanced distribution of prices, which reduced skewness and thereby compressed the impact of extreme values. A bimodal distribution is presented in the price column in the transformation. Tree-based models, which recursively divide feature spaces and isolate clusters using hierarchical partitions. Demir and Sahin (2024) argues that in machine learning models, choosing the most compatible data transformation method with the modeling technique leads to better results. In the end, the log-transformed price variable proved to be statistically more robust, significantly enhancing the reliability and accuracy of our modeling efforts.

The study found that, on average, pulses and nuts are the commodity categories with the highest average prices in the regions, which could be attributed to the nature of their production process or the scarcity of local resources required for their production. These food commodities are perishable and are produced seasonally. The case is in full agreement with Sturm and Datar (2011), which has shown that pulses typically experience higher market fluctuations due to storage and supply challenges in sub-Saharan Africa. The observation suggests that investments in these storage and preservation technologies are essential for price stabilization, as well as facilitating access, particularly for smallholder producers. This supports Kenya's established price stabilization strategies, such as the Cereals and Sugar Finance Corporation and stabilization funds, with the aim of insulating consumers against steep price hikes, which have often been defeated by poor storage capacity. In addition, this suggests that the recognition of buying habits during the week and on weekends parallels the consumer behavior observed by Waterlander et al. (2019), who found that, in general, price changes and demand surges are multiplied in time displacement throughout the week in developing markets. This research also revealed how fluctuations in exchange rates, particularly the continuous strengthening of the USD, influence commodity prices, primarily those that rely on foreign inputs. These commodities demonstrate a higher sensitivity to exchange rates, indicating Kenya's vulnerability to foreign exchange volatility. This situation represents not only the microeconomic aspects but also the macroeconomic aspects (Wang and Cheung, 2023). This vulnerability has led the government to implement agricultural input subsidies, such as the provision of fertilizer subsidy programs to farmers, to protect food systems from imported inflation, along with some foreign exchange interventions. This could lead to reduced affordability for households and create a string of pressures on the balance of payments and inflation. This, therefore, gives rise to the need to diversify local production, deepen regional trade, and develop import substitution policies (Wainaina, 2023; Titus et al., 2022).

Furthermore, the study also revealed that the price type has a high positive correlation with log-price-KES, with a magnitude of 0.70, implying that different pricing structures or schemes have a significant influence on price levelsÑa pattern consistent with the findings of Kozian et al. (2025). In contrast, the price flag shows a moderate negative correlation with the target variable (0.52), suggesting that the price flag, perhaps reflecting price brackets, varies inversely with log-price-KES (Kozian et al., 2025). Other categorical variables, such as commodity category and region, exhibit weak correlations with the target variable, suggesting that these classification factors have a less linear impact on price variations. This finding aligns with previous studies, which indicate weak linear impacts from such groupings (de Nicola et al., 2016; Deb et al., 1996). Numerical variables, such as the inflation rate, are weakly positively correlated, suggesting a very mild macroeconomic impact on the price structure. Most variables exhibit low pairwise correlations, indicating that there is no multicollinearity except for high correlations in price type and price flag. However, these predictor variables are statistically significant with *p*-values < 0.05, hence justifying the use of these predictors in tree-based models such as XGBoost, which are known to handle varying correlation structures effectively (Rabinowicz and Rosset, 2022).

Compared to standalone models, XGBoost delivered an extraordinarily high R^2^ of 0.9924, which is consistent with the results of Arora and Bansal (2019) in agricultural commodity prediction in India, where XGBoost surpassed random forest and decision tree regressors, as it employs regularization to avoid overfitting and is better at dealing with outliers. However, the hybrid model outperformed the standalone models, proving it to be the most effective model to use in this case. The model achieved metric scores of MAE 0.1152, MSE of 0.0290, RMSE of 0.1702, and an R^2^ of 0.9934, surpassing those of the individual models, thus confirming the advantages of hybrid ensemble learning. This aligns with the findings of Choudhary et al. (2025), which show that hybrid ensemble techniques achieve better results in food price prediction, as they can capture nonlinear features more effectively compared to single algorithms. However, the hybrid model was further improved through hyperparameter tuning, resulting in an MAE of 0.1050, an MSE of 0.0261, an RMSE of 0.1615, and an R^2^ of 0.9940. The R^2^ of 99.40% indicates that a variation in food price data is explained, with the model showing a significant reduction in prediction error. The results of this study are in line with those of Probst et al. (2019), who argued that hyperparameter tuning is a key factor in increasing the accuracy and robustness of a model, especially in economic data in real-world environments where noise is inevitable.

The results show that while standalone machine learning models such as random forest, decision tree, and LightGBM have higher training R^2^ scores of 0.9989, 0.9967, and 0.9964, respectively, the lower cross-validation scores of random forest (0.9290) indicate potential overfitting due to its feature of memorization of the patterns in the training data instead of generalization (Ghojogh and Crowley, 2023). In addition, while LightGBM achieves high levels of predictive accuracy and rapid training, its tendency toward a leaf-wise growth strategy makes it more prone to overfitting, particularly in cases of heterogeneous data (Ke et al., 2017). On the other hand, combining XGBoost and gradient boosting uses higher regularization, a balanced leaf growth pattern, and different learning biases to improve generalization and reduce error variance (Bentéjac et al., 2020). The current literature indicates that hybrid boosting approaches outperform standalone LightGBM or XGBoost for all datasets, as evidenced by lower error metrics, and yield stable predictions (Chen and Guestrin, 2016). Therefore, a hybrid model creates a more reliable method for robust food price prediction compared to LightGBM. Through stacking with linear regression as the meta-learner, the hybrid model combines the outputs from XGBoost and gradient boosting, yielding the best performance across all metrics, specifically cross-validated R^2^ with an average of 0.9931 and a standard deviation of 0.0011. Presumably, the hybrid method outperforms other models because it compensates for the shortcomings of conventional methods while leveraging their advantages, particularly by reducing variance while improving generalizability (Ragam et al., 2024; Wang et al., 2019). The results of the DM test in Table 10 show that, among all the best models, the hybrid model is statistically superior to the others at a significance level of 5%, which is also in accord with the results obtained by Qureshi et al. (2024). The stability of scores across all validation folds and the DM results further attest to the model's resilience and reliability, making it perhaps the best model for deployment in real-world predictions.

However, even though the hybrid model offers an excellent R^2^ (0.9940) and very low error metrics, the residual analysis reveals the danger of overfitting. The residual distribution indicates that, in general, while most predictions are close to the actual price, there is a systematic bias at the commodity level. Some commodity categories (2 and 7) exhibit relatively high variability (std >0.20) and wide residual ranges, indicating that the model does not effectively capture the volatility of these commodities. With some groups, such as category 4, the model has a consistent negative mean residual, while other groups, like category 2, consistently have positive mean residuals. Overall, this finding suggests that the model does very well, despite the shortage of datasets for some commodity categories. In these categories of consistent underestimation and overestimation, respectively, it may be possible to collect and add more datasets to improve the model's performance in these categories, as few records are available for the model to learn from. As noted in Choudhary et al. (2025), while hybrid ensemble models offer reasonable accuracy, they may still exhibit the same systematic biases and risk poorly adapting to sudden interference or market changes, underscoring the importance of conducting residual diagnostics and volatility-conscious model updates.

In addition, unit quantity and price type were found to be the most influential predictors of food prices, surpassing macroeconomic factors such as currency and inflation rates. The limited impact of exchange rate volatility on local commodity prices is consistent with the findings of Mwangi and Njoroge (2014) and Wanzala et al. (2024), both of whom demonstrated that such volatility has a negligible effect on food prices within countries, such as Kenya, that depend heavily on imported goods. This implies that local and transactional characteristics, such as pricing discrepancies between wholesale and retail markets or the behaviors of small-scale traders, have a high impact on commodity prices. These microeconomic factors may be better suited to representing short-term demand and supply relationships, as well as changes in the informal market, than more general economic trends. The micro effect of exchange rate fluctuations may also arise from the market's limited integration with international pricing forces. Corroborating these latter pieces of evidence, our exploration of linear feature importance plots using the top ten predictors showcased a better alignment between predicted and actual prices along the perfect prediction line, highlighting not only high modeling accuracy but also low prediction error.

The developed prediction model was then deployed. Generally, the system had four main components: user authentication and predictions, the back end that processes user requests by filling out the prediction form, the front end that displays the results, and the email message containing the prediction sent to the user. After analyzing feature importance, the top 10 features were selected based on their contribution to the model. The trained hybrid model (XGBoost and gradient boosting) was saved using the pickle module. The encoding object used during categorical and numerical feature transformation was also serialized. These files, including the model, feature list, and encoder, were crucial to ensure that new input data during deployment was transformed in the same way as the training data. This formed the foundation for deploying the model in a real-time environment.

The front-end features are designed using the Django templating system to provide users with a clean and intuitive interface. Users must register with their email and password to use the application, which involves a subsequent secure registration page. After registering, the user logs in and is directed to the web application's landing page, where details about the application, its purpose, and instructions on how to use it are provided. From this page, users can proceed to the prediction page, where a dynamic form will be generated with the top 10 identified features. Users are required to enter values pertinent to their food commodity and then submit the form to obtain predictions. The predicted price is displayed immediately on the page and also sent to the user's email. Sets were designed for usability and security enhancements. Validation was performed on both the client and server sides. The input must be valid; this, in turn, reduces the number of induced errors and increases the strength of the presented system. We also had exceptions handled well within the backend, such as when a user experienced a failed prediction or email delivery, and presumably, any other errors.

The backend was implemented in Python using the Django framework. When the server starts, the pre-trained model and encoder are loaded from serialized pickle files into memory. When the user inputs data from the front end, the application first validates the input data, applies the same encoding used during training, and passes it through the model to generate predictions. Since the model was trained on a log-transformed price variable, the predicted output is exponentiated to get back the actual price in KES. This allows the user to get meaningful and understandable price estimates. Once the prediction is complete, the price is sent to the registered user's email using Django's email system for convenience and record-keeping purposes.

In terms of system performance, the deployed model operates in real time, generating predictions and displaying them to the user within seconds of inputting the initial form. This is made possible by pre-loading the serialized model and encoder into memory when the server starts. By doing so, we have minimized the amount of latency when predictions are requested. Regarding error handling, we implemented error handling at various levels: we provided both client-side and server-side input validation, built-in exception handling to accommodate failed predictions, and issued alerts when emails failed to send, allowing users to be aware of any issues that may arise. Next, the system was designed to allow for periodic updates; the backend data and the model could be easily updated with new market data, the model retrained, and the web interface placed back into the hands of the user, allowing them to receive correct forecasts based on accurate and up-to-date market data.

In general, the application of machine learning models in predicting food prices has ethical implications. Being able to produce precise forecasts can significantly impact how markets behave, thereby creating speculative opportunities and even the potential for harm to more vulnerable groups when forecasts are misused. Several safeguards should therefore be introduced to mitigate the risks resulting from the investigation, such as transparent communication of the limitations of predictive models, the use of forecasts for policymaking and planning instead of profit manipulation, and equal access to knowledge. If embedded with ethical safeguards, such models can enable efficient decision-making without subsidizing market inequalities.

## 5 Conclusion

This study highlights the importance of food price prediction in Kenya, utilizing a hybrid machine learning approach that combines XGBoost and gradient boosting. The model shows robust performance compared to standalone models. The predictions generated by the model exhibit minimal errors and are closely aligned with the actual prices, indicating that it is unbiased. The results, therefore, support and expand the current conception of AI and its impact on agriculture and food systems. This demonstrates its ability to understand the price patterns of various commodities, thereby enhancing its reliability in real-world food price prediction applications. The findings of this work benefit a variety of sectors, including policymakers, agricultural stakeholders, and traders, providing a solid basis for understanding price trends in commodities and helping to inform strategic interventions to improve food security. In addition, this study provides a scalable framework that can be replicated by other Sub-Saharan countries. Although the research makes significant contributions, it also reveals notable research gaps that need to be addressed in future studies, such as combining socioeconomic data, customer behavior in terms of commodity purchase, and real-time data to enhance the accuracy of predictions. Coherently addressing these shortcomings in future studies could help improve the effectiveness and accuracy of predictions, leading to better decision-making and enhanced food security.

## Data Availability

The original contributions presented in the study are included in the article/supplementary material, further inquiries can be directed to the corresponding author.

## References

[B1] Abdallah M. B. Fekete-Farkas M. Lakner Z. (2021). Exploring the link between food security and food price dynamics: a bibliometric analysis. Agriculture 11:263. 10.3390/agriculture11030263

[B2] Abodi J. Aggrey-Nyachae J. Makokha M. (2021). Supply and demand responsiveness to maize price changes in Kenya. Cogent Food Agric. 7:1957318. 10.1080/23311932.2021.1957318

[B3] Abuzaid A. Alkronz E. (2024). A comparative study on univariate outlier winsorization methods in data science context. Italian J. Appl. Statist. 36, 85–99. 10.26398/IJAS.0036-004

[B4] Alam M. J. Parton K. P. Davidson S. R. (2014). The impacts of food price and income shocks on household food security and economic well-being: evidence from rural Bangladesh. Global Environ. Change 25, 150–162. 10.1016/j.gloenvcha.2014.02.003

[B5] Ali Y. A. Awwad E. M. Al-Razgan M. Maarouf A. (2023). Hyperparameter search for machine learning algorithms for optimizing the computational complexity. Processes 11:349. 10.3390/pr11020349

[B6] Arora R. Bansal P. (2019). XGBoost in agricultural commodity prediction: An empirical assessment. Comput. Agric. 8, 99–110.

[B7] Attílio L. A. (2024). Geopolitical risk and food price inflation: a GVAR analysis of Brics and the g7. Int. Trade J. 2024, 1–26. 10.1080/08853908.2024.2422119

[B8] Attobrah M. (2024). “Exploratory data analysis,? in *Essential Data Analytics, Data Science, and AI* (Berkeley, CA: Apress). 10.1007/979-8-8688-1070-1

[B9] benard3360-star (2025). Food_price: a web application for predicting agricultural commodity prices in Kenya using machine learning. GitHub repository. Available online at: https://github.com/benard3360-star/Food_price (Accessed August 26, 2025).

[B10] Bentéjac C. Csörgö A. Martínez-Muñoz G. (2020). A comparative analysis of gradient boosting algorithms. Artif. Intell. Rev. 54, 1937–1967. 10.1007/s10462-020-09896-5

[B11] Breiman L. (2001). Random forests. Mach. Learn. 45, 5–32. 10.1023/A:1010933404324

[B12] Central Bank of Kenya (2024). Forex exchange rates.

[B13] Chen T. Guestrin C. (2016). “Xgboost: a scalable tree boosting system,? in *Proceedings of the 22nd ACM SIGKDD International Conference on Knowledge Discovery and Data Mining* (ACM), 785–794. 10.1145/2939672.2939785

[B14] Choudhary J. Sharma H. K. Malik P. Majumder S. (2025). Price forecasting of crude oil using hybrid machine learning models. J. Risk Finan. Manag. 18:346. 10.3390/jrfm18070346

[B15] Choudhary K. Jha G. K. Jaiswal R. (2025). A genetic algorithm optimized hybrid model for agricultural price forecasting based on VMD and LSTM network. Sci. Rep. 15:9932. 10.1038/s41598-025-94173-0 40121306 PMC11929791

[B16] Dash C. S. K. Behera A. K. Dehuri S. Ghosh A. (2023). An outliers detection and elimination framework in classification task of data mining. Decis. Anal. J. 6:100164. 10.1016/j.dajour.2023.100164

[B17] de Nicola F. De Pace P. Hernandez M. A. (2016). Co-movement of major energy, agricultural, and food commodity price returns: a time-series assessment. Energy Econ. 57, 28–41. 10.1016/j.eneco.2016.04.012

[B18] Deb P. Trivedi P. K. Varangis P. (1996). The excess co-movement of commodity prices reconsidered. J. Appl. Econometr. 11, 275–291. 10.1002/(SICI)1099-1255(199605)11:3<275::AID-JAE392>3.0.CO;2-3

[B19] Demir S. Sahin E. K. (2024). The effectiveness of data pre-processing methods on the performance of machine learning techniques using RF, SVR, cubist and SGB: a study on undrained shear strength prediction. Stochast. Environ. Res. Risk Assess. 38, 3273–3290. 10.1007/s00477-024-02745-9

[B20] Diebold F. X. Mariano R. S. (1995). Comparing predictive accuracy. J. Bus. Econ. Statist. 13, 253–263. 10.1080/07350015.1995.10524599

[B21] Django Software Foundation (2025). Django: the web framework for perfectionists with deadlines. Available online at: https://www.djangoproject.com/ (Accessed July 23, 2025).

[B22] Dobbin K. K. Simon R. M. (2011). Optimally splitting cases for training and testing high dimensional classifiers. BMC Med. Gen. 4:31. 10.1186/1755-8794-4-31 21477282 PMC3090739

[B23] Ennaji O. Baha S. Vergutz L. El Allali A. (2024). Gradient boosting for yield prediction of elite maize hybrid zhengdan 958. PLoS ONE 19:e0315493. 10.1371/journal.pone.0315493 39689079 PMC11651618

[B24] Friedman J. H. (2001). Greedy function approximation: a gradient boosting machine. Ann. Stat. 29, 1189–1232. 10.1214/aos/1013203451

[B25] Ghojogh B. Crowley M. (2023). The theory behind overfitting, cross validation, regularization, bagging, and boosting: tutorial. arXiv preprint arXiv:1905.12787.

[B26] Gizaw D. Myrland O. (2025). Exploring the causes behind rising food prices in sub-saharan africa. Cogent Econ. Finan. 13:2461599. 10.1080/23322039.2025.2461599

[B27] He R. Li B. Li F. Song Q. (2024). A review of feature engineering methods in regression problems. Acad. J. Nat. Sci. 1, 32–40. 10.5281/zenodo.13905622

[B28] Headey D. D. Fan S. (2010). Reflections on the global food crisis: How did it happen? how has it hurt? And how can we prevent the next one? Research Monograph 165, International Food Policy Research Institute (IFPRI).

[B29] Heaton J. (2016). An empirical analysis of feature engineering for predictive modeling. SoutheastCon 2016, 1–6. 10.1109/SECON.2016.7506650

[B30] Iftikhar H. Mancha Gonzales S. Zywiolek J. López-Gonzales J. L. (2024). Electricity demand forecasting using a novel time series ensemble technique. IEEE Access 12, 88963–88975. 10.1109/ACCESS.2024.3419551

[B31] Iftikhar H. Qureshi M. Canas Rodrigues P. Usman Iftikhar M. Linkolk L?pez-Gonzales J. Iftikhar H. (2025). Daily crude oil prices forecasting using a novel hybrid time series technique. IEEE Access 13, 98822–98836. 10.1109/ACCESS.2025.3574788

[B32] International Monetary Fund (2024). Inflation rate - *Kenya (consumer prices)*. 10.5089/9798400285394.019

[B33] James G. Witten D. Hastie T. Tibshirani R. (2013). An Introduction to Statistical Learning: with Applications in R. Cham: Springer. 10.1007/978-1-4614-7138-7

[B34] Jayne T. Myers R. Nyoro J. (2006). “The effects of government maize marketing policies on maize market prices in Kenya,? in *International Association of Agricultural Economists, 2006 Annual Meeting* (Queensland, Australia).

[B35] Kalkuhl M. von Braun J. Torero M. (2015). The short-term impact of price shocks on food security: evidence from urban and rural Ethiopia. Food Secur. 7, 521–533. 10.1007/s12571-015-0467-4

[B36] Kalkuhl M. von Braun J. Torero M. (2016). “Volatile and extreme food prices, food security, and policy: an overview,? in Food Price Volatility and Its Implications for Food Security and Policy, eds. M. Kalkuhl, J. von Braun, and M. Torero (Cham: Springer), 3–31. 10.1007/978-3-319-28201-5_1

[B37] Katchali M. Senagi K. Richard E. Beesigamukama D. Tanga C. M. Athanasiou G.. (2024). Unveiling environmental influences on sustainable fertilizer production through insect farming. Sustainability 16:3746. 10.3390/su16093746

[B38] Kathiravelu P. Sharma A. Galhardas H. Van Roy P. Veiga L. (2018). On-demand big data integration: a hybrid ETL approach for reproducible scientific research. arXiv preprint arXiv:1804.08985. 10.1007/s10619-018-7248-y

[B39] Ke G. Meng Q. Finley T. Wang T. Chen W. Ma W.. (2017). “Lightgbm: a highly efficient gradient boosting decision tree,? in Advances in Neural Information Processing Systems (NeurIPS), 3146–3154.

[B40] Keene O. N. (1995). The log transformation is special. Stat. Med. 14, 811–819. 10.1002/sim.4780140810 7644861

[B41] Korir L. Rizov M. Ruto E. (2020). Food security in Kenya: insights from a household food demand model. Econ. Model. 92, 99–108. 10.1016/j.econmod.2020.07.015

[B42] Kozian L. L. Machado M. R. Osterrieder J. R. (2025). Modeling commodity price co-movement: building on traditional time series models and exploring applications of machine learning algorithms. Decis. Econ. Fin. 2025, 1–44. 10.1007/s10203-025-00512-1

[B43] Kunyanga C. N. Byskov M. F. Hyams K. Mburu S. Werikhe G. Bett R. (2023). Influence of COVID-19 pandemic on food market prices and food supply in urban markets in Nairobi, Kenya. Sustainability 15:1304. 10.3390/su15021304

[B44] Kyalo H. Tonnang H. Egonyu J. Olukuru J. Tanga C. Senagi K. (2025). Automatic synthesis of insects bioacoustics using machine learning: a systematic review. Int. J. Trop. Insect Sci. 45, 101–120. 10.1007/s42690-024-01406-2

[B45] Kyalo H. Tonnang H. E. Z. Egonyu J. P. Olukuru J. Tanga C. M. Senagi K. (2024). A convolutional neural network with image and numerical data to improve farming of edible crickets as a source of food—a decision support system. Front. Artif. Intell. 7:1403593. 10.3389/frai.2024.1403593 38808214 PMC11130480

[B46] Maharana K. Mondal S. Nemade B. (2022). A review: data pre-processing and data augmentation techniques. Global Trans. Proc. 3, 91–99. 10.1016/j.gltp.2022.04.020

[B47] Mamoudan M. M. Mohammadnazari Z. Ostadi A. Esfahbodi A. (2022). Food products pricing theory with application of machine learning and game theory approach. Int. J. Prod. Res. 62, 5489–5509. 10.1080/00207543.2022.2128921

[B48] Mastropaolo A. Aghajani E. Pascarella L. Bavota G. (2023). Automated variable renaming: are we there yet? Empir. Softw. Eng. 28:45. 10.1007/s10664-022-10274-8

[B49] Minot N. (2014). Food price volatility in sub-saharan Africa: has it really increased? Food Policy 45, 45–56. 10.1016/j.foodpol.2013.12.008

[B50] Muinde J. Tanga C. M. Olukuru J. Odhiambo C. Tonnang H. E. Z. Senagi K. (2023). Application of machine learning techniques to discern optimal rearing conditions for improved black soldier fly farming. Insects 14:479. 10.3390/insects14050479 37233107 PMC10231057

[B51] Mutwiri R. M. (2019). Forecasting of tomatoes wholesale prices of Nairobi in Kenya: time series analysis using Sarima model. Int. J. Statist. Distr. Applic. 5, 46–53. 10.11648/j.ijsd.20190503.11

[B52] Mwangi J. K. Njoroge E. W. (2014). Effects of exchange rate volatility on exports performance of French beans in Kenya. J. Bus. Manag. Econ. Res. 6, 91–107.

[B53] Nasir J. Iftikhar H. Aamir M. Iftikhar H. Rodrigues P. C. Rehman M. Z. (2025). A hybrid lmd-arima-machine learning framework for enhanced forecasting of financial time series: Evidence from the nasdaq composite index. Mathematics 13:2389. 10.3390/math13152389

[B54] Office for the Coordination of Humanitarian Affairs (2022). 47 counties of Kenya. Available onlinea at: https://data.humdata.org/dataset/47-counties-of-kenya (Accessed July 2, 2025).

[B55] Oztornaci B. Ata B. Kartal S. (2024). Analysing household food consumption in turkey using machine learning techniques. Econ. Inform. 16, 97–105. 10.7160/aol.2024.160207

[B56] Probst P. Wright M. N. Boulesteix A.-L. (2019). Hyperparameters and tuning strategies for random forest. WIREs Data Mining Knowl. Disc. 9:e1301. 10.1002/widm.1301

[B57] Pudjihartono N. Fadason T. Kempa-Liehr A. W. O'Sullivan J. M. (2022). A review of feature selection methods for machine learning-based disease risk prediction. Front. Bioinformat. 2:927312. 10.3389/fbinf.2022.927312 36304293 PMC9580915

[B58] Quirk T. J. Rhiney E. (2021). “Correlation and simple linear regression,? in *Excel 2019 for Marketing Statistics, Excel for Statistics* (Cham: Springer), 89–108. 10.1007/978-3-030-62781-2

[B59] Qureshi M. Iftikhar H. Rodrigues P. C. Rehman M. Z. Salar S. A. A. (2024). Statistical modeling to improve time series forecasting using machine learning, time series, and hybrid models: A case study of Bitcoin price forecasting. Mathematics 12:3666. 10.3390/math12233666

[B60] Rabinowicz A. Rosset S. (2022). Tree-based models for correlated data. J. Mach. Learn. Res. 23, 1–31. 10.48550/arXiv.2102.08114

[B61] Ragam P. Kushal Kumar N. Ajith J. E. Karthik G. Himanshu V. K. Sree Machupalli D.. (2024). Estimation of slope stability using ensemble-based hybrid machine learning approaches. Front. Mater. 11:1330609. 10.3389/fmats.2024.1330609

[B62] Sapakova S. Sapakov A. Madinesh N. Almisreb A. Dauletbek M. (2023). “Using machine learning to predict food prices in Kazakhstan,? in *Proceedings of the DTESI Workshops*. Short papers.

[B63] Senagi K. Jouandeau N. Kamoni P. (2017). Using parallel random forest classifier in predicting land suitability for crop production. J. Agric. Inform. 8, 23–32. 10.17700/jai.2017.8.3.390

[B64] Sharma S. Talavera L. Riquelme J. C. (2023). Encoding categorical data: Is there yet anything 'hotter' than one-hot encoding? *arXiv preprint arXiv:2312.16930*.

[B65] Shinyekwa I. Ijjo A. T. (2016). Determinants of domestic food price differentials: Constraints for intra-uganda trade. J. Sustain. Dev. 9:286. 10.5539/jsd.v9n1p286

[B66] Smith J. Johnson A. (2023). Data preprocessing: Identifying anomalies such as noise, inaccuracies, inconsistencies, and incompleteness in datasets. J. Data Sci. 5, 45–58. 10.1201/9781003284543-2

[B67] Sturm R. Datar A. (2011). Regional price differences and food consumption frequency among elementary school children. Public Health 125, 136–141. 10.1016/j.puhe.2010.11.016 21315395 PMC3073594

[B68] Tadesse G. Algieri B. Kalkuhl M. von Braun J. (2014). Drivers and triggers of international food price spikes and volatility. Food Policy 47, 117–128. 10.1016/j.foodpol.2013.08.014

[B69] Tesfaye W. Gebremariam G. (2020). Consumption smoothing and price enhancement motives for grain storage: empirical perspectives from rural Ethiopia. Agric. Food Econ. 8:25. 10.1186/s40100-020-00169-x

[B70] Titus O. M. Wagala A. Muriithi D. K. (2022). Foreign exchange rate volatility and its effect on international trade in Kenya. Eur. J. Human. Soc. Sci. 2, 47–56. 10.24018/ejsocial.2022.2.4.182

[B71] Ulussever T. Ertuğrul H. M. Kılıç Depren S. Kartal M. T. Depren M. (2023). Estimation of impacts of global factors on world food prices: a comparison of machine learning algorithms and time series econometric models. Foods 12:873. 10.3390/foods12040873 36832948 PMC9957413

[B72] Verma V. (2025). A comprehensive framework for residual analysis in regression and machine learning. J. Inf. Syst. Eng. Manag. 10, 1–18. 10.52783/jisem.v10i31s.4958

[B73] Vijayan V. Suresh A. Sabu S. Padi H. P. (2025). Fish for food and nutrition security in India: a comprehensive framework analysis. Food Secur. 17, 749–765. 10.1007/s12571-025-01532-w

[B74] Vos R. Jackson J. James S. SÃ¡nchez M. V. (2020). “Refugees and conflict-affected people: integrating displaced communities into food systems,? in *2020 Global Food Policy Report, chapter 5* (Washington, DC: International Food Policy Research Institute (IFPRI)), 46–53. 10.2499/9780896293670_05

[B75] Wainaina M. C. (2023). Impulse response to exchange rate, exports and imports shocks in Kenya 1990–2023. Int. J. Econ. 8, 1–15. 10.47604/ijecon.3150

[B76] Wang W. Cheung Y.-W. (2023). Commodity price effects on currencies. J. Int. Money Finan. 130:102745. 10.1016/j.jimonfin.2022.102745

[B77] Wang Y. Pan Z. Zheng J. Qian L. Li M. (2019). A hybrid ensemble method for pulsar candidate classification. Astrophys. Space Sci. 364:139. 10.1007/s10509-019-3602-4

[B78] Wanjuki T. M. Wagala A. Muriithi D. K. (2021). Forecasting commodity price index of food and beverages in Kenya using seasonal autoregressive integrated moving average (sarima) models. Eur. J. Mathem. Statist. 2, 50–63. 10.24018/ejmath.2021.2.6.80

[B79] Wanzala R. W. Marwa N. Nanziri Lwanga E. (2024). Impact of exchange rate volatility on coffee export in Kenya. Cogent Econ. Finan. 12:2330447. 10.1080/23322039.2024.2330447

[B80] Waterlander W. E. Jiang Y. Nghiem N. Eyles H. Wilson N. Cleghorn C.. (2019). The effect of food price changes on consumer purchases: a randomised experiment. Lancet Public Health 4, e394-e405. 10.1016/S2468-2667(19)30105-7 31376858

[B81] Were V. Foley L. Musuva R.. (2023). Socioeconomic inequalities in food purchasing practices and expenditure patterns: results from a cross-sectional household survey in western Kenya. Front. Public Health 11:943523. 10.3389/fpubh.2023.943523 36778539 PMC9909229

[B82] West R. M. (2022). Best practice in statistics: the use of log transformation. Ann. Clin. Biochem. 59, 162–165. 10.1177/00045632211050531 34666549 PMC9036143

[B83] World Food Programme (2025). WFP Food Prices for Kenya. Available online at: https://data.humdata.org/dataset/wfp-food-prices-for-kenya (Accessed October 10, 2025).

[B84] Zhang N. An Q. Zhang S. Ma H. (2025). Price prediction for fresh agricultural products based on a boosting ensemble algorithm. Mathematics 13:71. 10.3390/math13010071

